# Zinc oxide nanoparticles against *Candida*: mechanistic pathways, synergistic potentials, and biocompatibility challenges in the era of antimicrobial resistance

**DOI:** 10.3389/frabi.2026.1933663

**Published:** 2026-09-11

**Authors:** Binaya Krushna Sahu, Tapan Kumar Mohanta, Mahesh Chandra Sahu, Sujogya Kumar Panda

**Affiliations:** 1 Centre for Biotechnology, Siksha ‘O’ Anusandhan (Deemed to be University), Kalinganagar, Bhubaneswar, Odisha, India; 2 Dept. of Genetic Research, Institute for Research and Medical Consultations, Imam Abdulrahman Bin Faisal University, Dammam, Saudi Arabia; 3 Division of Microbiology, ICMR-Regional Medical Research Centre, Chandrasekharpur, Bhubaneswar, Odisha, India

**Keywords:** antifungal activity, biofilm inhibition, *Candida* species, drug resistance, green synthesis, mechanism of action, zinc oxide nanoparticles

## Abstract

The emergence of antifungal resistance among diverse *Candida* species highlights the need for a more efficient and effective approach to address this issue. Nanotechnology has emerged as a transformative approach with great promise to counter antifungal resistance, a global concern that has surged over the past two decades. Antifungal activity of zinc oxide nanoparticles (ZnO NPs), their green synthesis approaches, and mechanisms of action are promising characteristics that warrant systematic investigation. This review discusses the current literature available on ZnO NPs focusing on the approaches used in the synthesis of these nanoparticles, characterization, antifungal efficacy of these nanoparticles against *Candida* species, mechanisms of action, and effects on biofilm formation, potential synergistic effects with antifungal compounds, as well as their biocompatibility. From 324 PubMed and 688 Scopus records, 44 and 66 articles, respectively, were screened; after exclusions, 33 original studies (20 from Scopus and 13 from PubMed) were included in this review. Based on reviewed studies, ZnO NPs demonstrate antifungal activity against several *Candida* species, such as *Candida albicans*, *C. glabrata, C. auris, C. tropicalis, C. parapsilosis, C. krusei*, and *C. famata*, and the possible mechanisms of action of ZnO NPs are reactive oxygen species production, release of Zn²^+^ ions, cell membrane disruption, oxidative stress induction, inhibition of biofilm formation, and suppression of virulence genes. The green synthesis of ZnO NPs exhibited all but one of the traits required for biocompatibility. Their combination with standard antifungals enhanced antifungal activity. ZnO NPs therefore represent a promising antifungal strategy for the treatment of candidiasis. To ensure safety and therapeutic applicability, however, additional validation through standardized research, pharmacokinetic and toxicological evaluations, and well-designed clinical trials is necessary.

## 1 Introduction

The burden of fungal infection has emerged as an increasingly important global public health concern. Opportunistic fungal infections are of particular significance in immunocompromised individuals, intensive care unit patients, organ transplant recipients, and those with prolonged use of antimicrobial agents. *Candida* species are the main causative agents of most opportunistic infections, ranging from superficial mucosal infections to life-threatening invasive fungal infections (Bongomin et al., 2017). The major fungal pathogen causing fungal disease is *Candida albicans*. However, infections caused by non-albicans *Candida*, such as *C. glabrata*, *C. tropicalis*, *C. parapsilosis*, and *C. krusei*, are increasingly prevalent, as well as the emergence of multidrug-resistant (MDR) *C. auris* isolates (Katsipoulaki et al., 2024; Lockhart et al., 2017). *Candida* species harbor several virulence factors to adhere to host tissues, convert from yeast to hyphae, form biofilms, release hydrolytic enzymes and evade immune responses (Rodríguez-Cerdeira et al., 2020).

The growing number of immunocompromised patients and the increasing number of invasive procedures performed are among the factors responsible for the persistent increase in the global burden of candidiasis worldwide. The mortality rate of invasive candidiasis remains as high as 30-60%, despite improvements in its treatment and management (Martin-Loeches et al., 2025; Sahu et al., 2026). The increasing prevalence of healthcare-associated infections due to *Candida* species is not only placing a substantial economic burden on healthcare systems but also prolonging patients’ hospital stays. The yeast *C. auris* is an emerging pathogen that has evolved into a significant global public health threat worldwide over the past decade. Its ability to strongly adhere to and persist on environmental surfaces represents an important pathogenic characteristic, making it challenging to eradicate once established in healthcare settings (Panda et al., 2023).

Currently available antifungals: azoles, polyenes, echinocandins, and pyrimidine analogues are the four primary categories of antifungal medications that target the fungal cell membrane, interfere with nucleic acid metabolism, and prevent the formation of cell walls. The use of these antifungals has significantly improved the treatment of candidiasis (Carmo et al., 2023; Debta et al., 2026; Mallick et al., 2025). Nevertheless, some toxic effects of these drugs, limited antifungal action on some fungi, inadequate penetration into infected tissues, potentially harmful drug interactions, long period of treatment, and the rapid development of resistance to antifungals will continue to limit the use of these drugs clinically (Carmo et al., 2023; Lee et al., 2020). *Candida* develops resistance through gene mutation, increased efflux pump activity, alterations in membrane sterols, activation of stress response, and formation of highly tolerant biofilms (Debta et al., 2026; Murphy and Bicanic, 2021; Taff et al., 2013). Because biofilms have been shown to have antibiotic resistance levels 1000 times higher than those of their planktonic counterparts, biofilm formation on medical devices such as catheters, prosthetic heart valves, dentures, and prosthetic implants is especially concerning (Chandra et al., 2012; Ramage et al., 2006).

However, the rising rate of antifungal resistance and its adverse effects on humans and the environment have prompted scientists to explore alternative treatments to overcome resistance in *Candida* species. The science of nanotechnology has emerged as a promising interdisciplinary approach for developing antimicrobial products (de Almeida Campos et al., 2025; Gao et al., 2024). Scientists can utilize the unique physical and chemical properties of metal nanoparticles, such as their nanoscale size, high surface area to volume ratio, high surface reactivity, adaptability to potential functionalization, and the versatile nature of their biological actions, by using a variety of metal nanoparticle types. Nanoparticles can be employed as antifungal agents in this manner (Cruz-Luna et al., 2021; Nami et al., 2021; Wu et al., 2025).

The process of obtaining metal oxide nanoparticles via green synthesis using plant extracts has become a viable means of producing nanoparticles compatible with living organisms. The latest findings demonstrate that Zinc oxide nanoparticles (ZnO NPs) and TiO_2_ nanoparticles synthesized with the help of plants display high antioxidant, antimicrobial, and antifungal properties, caused by the synergistic action of plant chemicals and properties of nanoparticles themselves (Tiwari et al., 2024a, b; Tripathi et al., 2025). Additionally, the research confirms that altering the physical and chemical properties of nanoparticles can improve their effectiveness against microorganisms (Singh et al., 2025). Thus, one can conclude that ZnO NPs obtained via green synthesis can be effectively utilized to treat candidiasis with a positive impact on the human organism (Tiwari et al., 2026).

ZnO NPs have gained widespread popularity for their broad-spectrum antimicrobial activity, good chemical stability, strong photocatalytic properties, and biocompatibility with cells (El-Saadony et al., 2024). ZnO NPs exert their antifungal effects through multiple mechanisms that may help limit the development of resistance. Reactive oxygen species (ROS) production, fungal cell wall and membrane disruption, oxidative stress, mitochondrial dysfunction, DNA damage, protein oxidation, and metabolic inhibition are among the actions (Abou-Dobara et al., 2025; Hosseini et al., 2020; Shoeb et al., 2013). Moreover, ZnO NPs can inhibit *Candida* species adhesion to surfaces, prevent filamentous growth, inhibit quorum sensing, and prevent biofilm formation (Joshi et al., 2022). Thus, the multimodal action of ZnO nanomaterials will distinguish them from many common antifungal compounds that target only a single cellular pathway.

The development of eco-friendly methods for synthesizing ZnO NPs is another positive aspect of these nanoparticles. Common methods of chemical or physical synthesis of nanomaterials involve toxic reducing agents, volatile organic solvents, high temperatures, high energy inputs, and the formation of environmental by-products. Unlike traditional methods, green synthesis synthesizes ZnO NPs under mild conditions and produce less harmful waste by using reducing and stabilizing agents derived from plant extracts as well as bacteria, fungi, and algae (Mohammed et al., 2024; Sharma and Ghose, 2015; Souza et al., 2020; Sun et al., 2018). Moreover, due to the use of biogenic ZnO NPs obtained through these processes, they exhibit higher biological activities since these nanoparticles possess additional properties as a result of the presence of phytochemicals or metabolites on the surface of biogenic ZnO NPs. The synthesis methods mentioned above comply with the principles of green chemistry, and their successful use of these methods in ZnO NP synthesis contributes to the development of safer nanomaterials for medical applications.

According to Fayed et al. (2025), chemically synthesized ZnO NPs exhibited significantly greater antifungal efficacy against *C. auris* than biologically synthesized, with lower MIC values and robust inhibition of biofilm formation. While chemically synthesized ZnO NPs did not upregulate antifungal resistance genes, biologically synthesized ZnO NPs effectively inhibited fungal adherence and reduced the expression of key biofilm-associated genes (Fayed et al., 2025). Additionally, biologically synthesized ZnO NPs have a synergistic effect with the traditional antifungal agent fluconazole and show promise for combination therapy against *C. albicans* and *C. parapsilosis* (Yassin et al., 2025a). Furthermore, the therapeutic applications of ZnO NPs extend to include dentistry, implant coatings, wound dressings, drug delivery systems, and other medical devices.

These achievements emphasize the significance of understanding the antifungal properties of ZnO NPs, their safe synthesis, their potential biomedical applications, the associated safety issues, and the challenges of translating them to human applications. This review will provide an overview of green synthesis approaches for ZnO NPs, some of their physical and chemical properties, their mechanism(s) of action against *Candida* species, how they can be used as antifungal agents in combination with other antifungal agents, what is known about their toxicity, and prospects for future application. By synthesizing recent advances in nanotechnology, microbiology, and therapeutics for fungal infections, this paper presents a comprehensive overview of the potential uses of ZnO NPs as safe, effective treatments for candidiasis and as a means to address the global problem of antifungal resistance. The general antimicrobial applications of ZnO NPs have been highlighted in previous reviews; still, none have thoroughly discussed their mechanisms of action specific to *Candida*, their synergistic interactions with conventional antifungal drugs, or their biocompatibility in connection to antimicrobial resistance. Thus, this review discusses future approaches for the clinical translation of ZnO NP based antifungal medicines, identifies existing knowledge gaps, and offers an updated critical synthesis of recent findings. ‘

## 2 Methodology

### 2.1 Literature search strategy

This review aimed to conduct a comprehensive search of peer-reviewed literature regarding the ZnO NPs and their antifungal efficacy against *Candida* species. The literature search was performed using two major scientific databases, Scopus and PubMed, and was restricted to English language publications. To develop a search strategy, combinations of Medical Subject Headings (MeSH), keywords, and Boolean operators were used to ensure that a large number of relevant publications would be identified. The Boolean search string used was (‘zinc oxide nanoparticles’ or ‘ZnO nanoparticles’) and (*‘Candida’* or *‘Candida albicans*’ or *‘Candida auris’*). In total, 324 records from PubMed and 688 records from Scopus were found in the first literature search. Of these, 44 and 66 articles, respectively, were chosen for full-text evaluation after titles and abstracts were screened. The eligibility analysis detected duplicate records, review articles, inaccessible full-text publications and studies that did not meet the predefined inclusion criteria. In the end, 33 original research articles were included in the qualitative synthesis of this review after 20 studies from Scopus and 13 from PubMed met all eligibility criteria. The studies published from January 2010 to May 2026 were considered for this review.

### 2.2 Inclusion criteria

Studies were considered eligible for inclusion if they fulfilled the following criteria: (a) original, peer-reviewed research papers, (b) studies of the antifungal activity of ZnO NPs against *Candida* species, (c) investigations that included synthesis, characterization, and/or biomedical applications of ZnO NPs with antifungal relevance, (d) Studies of mechanisms of action of antifungal ZnO NPs and the generation of ROS, disruption of membranes, antibiofilm activity or synergistic effects, (e) studies carried out either *in vitro*, *in vivo*, or ex vivo, and (f) complete articles published in English.

### 2.3 Exclusion criteria

If a literature search meets at least one of the exclusion criteria that follow, all literature will be excluded from the review process: (a) The literature is a review article, systematic review, meta-analysis, conference abstract, editorial letter, book chapter, dissertation, or thesis, (b) The study examined nanoparticles other than ZnO and did not include ZnO NPs, (c) The publication is unrelated to *Candida*, (d) The study examined antimicrobial (antibacterial, antiviral, antiparasitic) activity, (e) The article does not contain experimental data on antifungal activity, (f) The literature is not published in English language.

### 2.4 Data extraction and synthesis

All relevant data were collected from each eligible study using a standardized data-collection format and systematic methods. Data that was collected: included study, types of *Candida* species used in the investigation, type of ZnO NPs method used, physicochemical characteristics of the ZnO NPs created, experimental models used, antifungal effectiveness demonstrated with ZnO NPs as a treatment against *Candida* species, ability to inhibit biofilm formation using ZnO NPs, proposed mechanisms of action for ZnO NPs as antifungal agents, synergistic interactions with other antifungal agents, evidence of cytotoxicity, and a summary of the overall findings based on the study infection. The corresponding studies were then compiled into thematic categories to perform qualitative synthesis and identify studies demonstrating current supporting evidence that ZnO NPs are an environmentally responsible antifungal method against *Candida* species.

## 3 Green synthesis of ZnO NPs

Green synthesis overcomes the drawbacks of traditional physical and chemical synthesis methods, which rely on hazardous chemicals, high energy consumption, and toxic solvents, to produce ZnO NPs in an environmentally friendly and sustainable manner (Sharma and Ghose, 2015; Souza et al., 2020; Mohanta et al., 2023). This method converts zinc salts into ZnO NPs under mild reaction conditions by using biological resources such as plant extracts, bacteria, fungi, algae, proteins, and polysaccharides as natural reducing, capping, and stabilizing agents. Because they include bioactive substances such flavonoids, phenolics, alkaloids, terpenoids, and proteins that improve stability and biological activity while facilitating nanoparticle formation, plant extracts are especially appealing (Shoeb et al., 2013; Souza et al., 2020). ZnO NPs with promising antimicrobial and antifungal properties have been successfully biosynthesized from a variety of plant species, including *Trigonella foenum-graecum*, *Camellia sinensis*, *Salvia officinalis*, *Mangifera indica*, *Citrus limon*, and *Anacardium occidentale*, as well as microorganisms, including *Pseudomonas putida*, *Lactobacillus gasseri*, and *Bacillus licheniformis* (Metwally et al., 2022; Yassin et al., 2023a, b; Mohammed et al., 2024; Abou-Dobara et al., 2025; Anitha et al., 2023; Fayed et al., 2025; Mathew et al., 2025; Yassin et al., 2025a).

Precursor concentration, pH, reaction temperature, extract composition, and calcination conditions all affect the physicochemical properties of green-synthesized ZnO NPs, including particle size, morphology, crystallinity, and surface charge (Sharma and Ghose, 2015; Mohammed et al., 2024). Because phytochemicals or microbial metabolites remain adsorbed on the nanoparticle surface, thereby providing additional antioxidant and antibacterial properties while reducing cytotoxicity, green-synthesized ZnO NPs often exhibit improved biocompatibility (Souza et al., 2020; Metwally et al., 2022). Additionally, several studies have shown that these nanoparticles have strong antifungal activity against *Candida* species and can work in concert with traditional antifungal medications like amphotericin B and fluconazole, underscoring their potential as safe and efficient nanotherapeutic agents (Mathew et al., 2025; Yassin et al., 2025a; Abou-Dobara et al., 2025).

## 4 Mechanisms of action of ZnO NPs against *Candida*

ZnO NPs have multiple mechanisms of action that contribute to their antifungal activity and are therefore attractive alternative agents to traditional antifungal therapies. ROS-induced oxidative stress was the primary mechanism proposed in the articles included in this review. Membrane damage, Zn^2+^ ion release, mitochondrial dysfunction, inhibition of biofilm formation, and modulation of virulence-associated gene expression were also reported (Aslani et al., 2018; Reda and Fayed, 2023; Sharma and Ghose, 2015; Shoeb et al., 2013; Yassin et al., 2025b). The broad-spectrum efficacy of ZnO NPs against both susceptible and multidrug-resistant *Candida* isolates is probably due to these multifaceted effects targeting multiple cellular processes, which also reduce the likelihood of resistance development. The characteristics of the included studies, synthesis methods, nanoparticle properties, and characterization techniques are summarized in **Table 1**. **Supplementary Table 1** summarizes the antifungal efficacy of ZnO NPs, including antifungal test results, MIC/MFC values, biofilm inhibition, mechanisms of action, synergistic effects with antifungal medications, cytotoxicity, and key findings.

**Table 1:** Characteristics of the included studies on zinc oxide nanoparticles against *Candida* species, including study details, nanoparticle synthesis, biological source, physicochemical properties, and characterization methods.

Reference	Country	*Candida* species studied	ZnO NP synthesis method	Biological source (green synthesis)	Particle size (nm)	Particle shape	Characterization methods
(Abedzadeh et al., 2022)	Iran	*C. albicans* (fluconazole-resistant isolates from vulvovaginal candidiasis)	Wet chemical synthesis using zinc chloride and sodium hydroxide	–	35 nm	Spherical, white, rough-surfaced aggregates	XRD, SEM
(Abou-Dobara et al., 2025)	Egypt	*C. albicans* ATCC 10231	Green synthesis (intracellular bacterial biosynthesis of ZnO NPs followed by fabrication of ZnO/chitosan/vancomycin nanocomposite)	*Bacillus licheniformis* ATCC 4527	79.38 nm (XRD); 78 ± 2.3 nm (TEM)	Spherical	UV-Vis, FTIR, XRD, TEM, Zeta potential
(Ahmadpour Kermani et al., 2021)	Iran	*C. albicans, C. krusei, C. parapsilosis, C. tropicalis*, and *C. lusitaniae*	Commercially purchased ZnO NPs (Iranian Nanomaterial Pioneer Co., Mashhad, Iran); no synthesis performed in this study	–	ZnO NPs: 10-30 nm (average ~20 nm); TiO_2_ NPs: 10-25 nm	ZnO: Spherical; TiO_2_: Spherical and rod-shaped	SEM, UV spectroscopy, XRD
(Albuquerque et al., 2023)	India	*C. albicans* (ATCC 14053)	Comparative study of chemically synthesized ZnO NPs (CSZnO-NPs) and green synthesized ZnO NPs (BSZnO-NPs) incorporated into soft denture liner	*Azadirachta indica* (Neem) leaf extract	CSZnO-NPs: ~71 nm; BSZnO-NPs: <100 nm	Spherical with an irregular rough surface	SEM, UV-Visible spectroscopy, FTIR, XRD
(Alves et al., 2018)	Portugal	*C. albicans* (SC5314) and *C. parapsilosis* (ATCC 22019)	Physical/electrochemical synthesis (electrodeposition of ZnO rod-like coating on Zn substrate)	–	Microstructured ZnO rod-like coating; exact particle size not reported	Rod-like ZnO crystals grown on hexagonal ZnO crystals (microstructured)	SEM, TEM, EDS, BSE
(Anitha et al., 2023)	India	*C. glabrata* (fluconazole-resistant clinical isolates)	Plant-mediated biosynthesis	*Anacardium occidentale* (cashew apple juice)	155.12 nm (XRD); 106.7-169.2 nm (SEM)	Flower-shaped (nanoflower), petal-like polycrystalline aggregates	UV-Vis, FTIR, XRD, SEM
(Aslani et al., 2018)	Iran	*C. albicans* ATCC 10231 and fluconazole-resistant clinical isolate	Chemical synthesis (ZnO NPs coated with chitosan-linoleic acid)	–	–	–	qRT-PCR, MTT assay, ROS assay (DCFH-DA), ELISA-based biofilm assay
(Dananjaya et al., 2018)	Republic of Korea	*C. albicans*	Chemical synthesis of ZnO NPs followed by fabrication of ZnO-chitosan nanocomposites (ZnO-C NCs)	–	ZnO NPs: ~35-40 nm; ZnO-chitosan nanocomposites: ~50–70 nm (agglomerated clusters)	Predominantly spherical particles forming clustered nanocomposites	XRD, FESEM, TEM, EDS, UV-Visible spectroscopy
(Das et al., 2016)	India	*C. krusei*	Chemical synthesis of Ag@ZnO core-shell nanocomposites	–	Ag core: ~17 nm; Ag@ZnO core-shell nanocomposite: ~65 nm	Spherical core-shell nanoparticles	XRD, FESEM, TEM, EDX, UV-Visible spectroscopy, FTIR, Photoluminescence (PL), Zeta potential
(Djearamane et al., 2022)	Malaysia (collaborative study with India)	*C. albicans* ATCC 10231	Commercial ZnO NPs (<100 nm; Sigma-Aldrich); no synthesis performed by the authors	–	59.1 nm (average crystallite/particle size)	Predominantly spherical nanoparticles with a hexagonal wurtzite crystalline structure	SEM, SEM-EDX, XRD, FTIR
(Fayed et al., 2025)	Egypt	*C. auris*	Comparative study of biologically synthesized ZnO NPs (ZnO-NP-B) using *Lactobacillus gasseri* and chemically synthesized ZnO NPs (ZnO-NP-C1, dry-wet chemical method; ZnO-NP-C2, sol–gel method)	*Lactobacillus gasseri* (for biological synthesis); Not applicable for chemical synthesis	ZnO-NP-C1: ~23 nm; ZnO-NP-C2: ~82 nm (nanoflakes); ZnO-NP-B: ~116 nm	ZnO-NP-C1: spherical; ZnO-NP-C2: nanoflake/plate-like; ZnO-NP-B: irregular spherical aggregates	Particle Size Analyzer (PSA), Zetasizer (photon correlation spectroscopy), SEM, XRD (ZnO-NP-C2), Zeta potential analysis, RT-qPCR
(Fozouni and Tahaei, 2023)	Iran	Clinical isolates of *C. albicans* (n = 41) and *C. famata* (n = 8) recovered from diarrheic calves; fluconazole-resistant isolates were evaluated for ZnO NP susceptibility.	Chemical synthesis using zinc acetate dissolved in deionized water, followed by heating at 100 ± 5°C for 12 h and calcination at 300 ± 20°C for 24 h to obtain ZnO nanoparticle crystals.	–	20 nm (TEM diameter)	Not explicitly reported (TEM image only)	TEM and XRD; UV-Visible spectroscopy was used to confirm nanoparticle formation.
(Hameed et al., 2015)	India	*C. albicans* (ATCC 10231)	Pure and Mg²^+^, Ca²^+^, Sr²^+^, and Ba²^+^-doped ZnO NPs synthesized by the co-precipitation method using zinc nitrate, metal nitrates, NaOH, and PEG.	–	Crystallite size (XRD): Pure ZnO = 38 nm; ZnO : Mg = 31 nm; ZnO : Ca = 37 nm; ZnO : Sr = 23 nm; ZnO : Ba = 28 nm.	Nanoflake morphology; Mg-doped ZnO showed aggregated nanoflakes with rough surfaces.	XRD, HRSEM (FESEM), EDAX, Zeta potential analysis.
(Hosseini et al., 2018)	Iran	*C. albicans* (113 fluconazole-susceptible and 20 fluconazole-resistant urinary catheter isolates; ATCC 10231 used as reference)	ZnO NPs synthesized by the sol-gel method using zinc acetate in gelatin medium, producing a hexagonal wurtzite structure.	–	20-50 nm (approximately 30 nm)	Spherical, with low agglomeration	XRD, SEM, CV assay, CFU enumeration
(Hosseini et al., 2019)	Iran	Clinical *C. albicans* isolates from urinary tract infections (UTIs), including fluconazole-resistant strains.	Chemical synthesis (zinc acetate precursor)	–	20-40 nm	Spherical	SEM, XRD, Real-time PCR, MTT assay
(Hosseini et al., 2020)	Iran	Clinical *C. albicans* isolates from women with vulvovaginal candidiasis (196 isolates; 10 fluconazole-resistant isolates evaluated for gene expression)	Commercial ZnO NPs were used for antifungal evaluation; the study did not synthesize nanoparticles.	–	–	–	No nanoparticle characterization was performed (commercial ZnO NPs were used). Identification of isolates by ITS-PCR; fluconazole susceptibility by CLSI disk diffusion; ERG11 detection by RT-PCR; SAP1–SAP3 expression by quantitative real-time PCR.
(Jalal et al., 2018)	India	Clinical isolates of *C. albicans*, *C. tropicalis*, and *C. dubliniensis*	Green synthesis	*Crinum latifolium* leaf extract	20-50 nm	Predominantly spherical	UV-Visible spectroscopy, FTIR, XRD, SEM, TEM, EDX
(Joshi et al., 2022)	India	*C. albicans*	Green synthesis using lignin-derived biotemplates (lignin, fragmented lignin [FL], and oxidized fragmented lignin [OFL]) to produce hierarchical ZnO NPs	Lignin, fragmented lignin (FL), and oxidized fragmented lignin (OFL)	≈40 nm (average particle diameter)	Hierarchical one-dimensional arrays of hexagonal wurtzite ZnO NPs	XRD, FESEM, TEM, FTIR, UV-Visible spectroscopy, RT-PCR, CLSM, Flow cytometry, MTT assay
(Jothiprakasam et al., 2017)	India	*C. tropicalis* (ATCC 13803, fluconazole-susceptible; ATCC 750, fluconazole-resistant)	Green synthesis (egg albumin-mediated biosynthesis using zinc acetate)	Egg albumin	5-50 nm	Spherical, agglomeration-free	UV-Visible spectroscopy, TEM
(Katafa and Hamid, 2020)	Iraq	*C. albicans*	Chemical synthesis (precipitation method; calcined and non-calcined ZnO nanoparticles)	–	30 nm (calcined), 28 nm (non-calcined) by TEM; 38 nm (calcined), 37 nm (non-calcined) by XRD crystallite size	Spherical	XRD, TEM, AFM
(Maniah, 2023)	Saudi Arabia	*C. albicans, C. glabrata, C. parapsilosis, C. tropicalis*, and *C. auris*	Green synthesis (co-precipitation method)	*Laurus nobilis* (bay leaf) aqueous extract	37.98 nm (average particle size)	Hexagonal crystalline ZnO NPs	UV-Visible spectroscopy, FTIR, XRD, TEM, DLS, Zeta potential, EDX, SEM
(Mathew et al., 2025)	India	*C. albicans*	Green synthesis followed by one-pot Amphotericin B conjugation	*Mangifera indica* (mango) leaf extract	21-83 nm (TEM, green ZnO NPs); 10-21 nm (AmB-conjugated ZnO NPs); hydrodynamic size by DLS: 1043.1 nm (ZnO NPs) and 936.8 nm (AmB@ZnONPs)	Irregular spherical (green ZnO NPs); smaller surface-functionalized particles after Amphotericin B conjugation	UV-Vis, FTIR, XRD, SEM, TEM, DLS, Zeta potential, TGA/DTA
(Metwally et al., 2022)	Egypt	Two clinical isolates of *C. albicans*, *C. glabrata*, and *C. krusei* (MDR clinical isolates)	Green synthesis (plant-mediated)	*Citrus limon* (lemon peel aqueous extract)	13.58-30.70 nm (HR-TEM); 98.77 nm hydrodynamic size (DLS)	Rod-shaped	UV-Visible spectroscopy, HR-TEM, DLS, Zeta potential analysis
(Mohammed et al., 2024)	Iraq	*C. albicans* (clinical isolates from leukemia patients)	Green synthesis (microbial biosynthesis)	*Pseudomonas putida* culture filtrate	20-40 nm (XRD); 69 nm (AFM average)	–	AFM, XRD, FTIR, SEM, TEM, XPS
(Padalia and Chanda, 2017)	India	*C. albicans*, *C. glabrata* (clinical isolates of *Candida* spp.); *Cryptococcus neoformans* was also evaluated	Green synthesis	*Ziziphus nummularia* leaf extract	12.47-26.97 nm (average 17.33 nm)	Predominantly spherical with some irregular-shaped nanoparticles	UV-Visible spectroscopy, FTIR, XRD, TEM, SAED
(Reda and Fayed, 2023)	Egypt	*C. auris* (CDC-B11903 and CDC-CAU9; MDR strains)	Chemical synthesis (sol-gel citrate route) using calcium-doped ZnO ceramic NPs [(1−x) ZnO–xCaO]	–	367 ± 50.99 nm (ZC0), 226.9 ± 29.18 nm (ZC10), 137.3 ± 8.16 nm (ZC90)	Nanoflake-like (ZC0); regular hexagonal (Ca-doped ZC10 & ZC90)	XRD, SEM, DLS (Zetasizer), Zeta potential
(Sharma and Ghose, 2015)	India	*C. albicans* (MTCC 221)	Chemical synthesis (homogeneous precipitation method without surfactant, chelating, or gelling agents)	–	30.6 ± 0.3 nm (300°C) and 33.8 ± 0.5 nm (400°C) by TEM; crystallite size 29.4-31.7 nm by XRD	Agglomerated spherical particles with uniform hexagonal morphology after calcination at 400°C (rod-like morphology before calcination)	XRD, FTIR, TGA, UV-Vis diffuse reflectance spectroscopy, BET surface area analysis, FE-SEM, EDXA, TEM
(Shoeb et al., 2013)	India	*C. albicans* 077 (clinical MDR isolate)	Green synthesis (biotemplate-assisted synthesis)	Egg albumen (egg white)	10-20 nm (TEM); ~16 nm (XRD crystallite size); 8-22 nm (AFM)	Spherical, polycrystalline hexagonal wurtzite ZnO	XRD, TEM, AFM, EDAX, FTIR, UV-Vis spectroscopy, Fluorescence spectroscopy, TGA/DTA, SEM
(Souza et al., 2020)	Brazil	*C. albicans* ATCC 18804, *C. krusei* ATCC 6258, *C. parapsilosis* ATCC 22019, *C. tropicalis* ATCC 750	Green synthesis using natural polysaccharides (cashew gum and carboxymethylated cashew gum) as stabilizing/capping templates	Cashew gum (CG) and Carboxymethylated Cashew Gum (CMCG) obtained from *Anacardium occidentale*	35-129 nm (AFM); crystallite size 27.7-31.7 nm (XRD)	Round-shaped (spherical) ZnO NPs with a hexagonal wurtzite crystalline phase	UV-Vis spectroscopy, FTIR, XRD, AFM
(Yassin et al., 2023a)	Saudi Arabia	*C. albicans*, *C. glabrata*, *C. tropicalis*	Green synthesis	*Salvia officinalis* (sage) leaf aqueous extract	12.9 nm (XRD); 27.9 ± 3.7 nm (TEM); hydrodynamic diameter 83.46 nm (DLS)	Hexagonal wurtzite crystalline nanoparticles	UV-Visible spectroscopy, FTIR, XRD, TEM, DLS, Zeta potential (−8.3 mV), EDX, SEM
(Yassin et al., 2023b)	Saudi Arabia	*C. albicans*, *C. tropicalis*, *C. glabrata*	Green synthesis	*Camellia sinensis* (green tea) leaf aqueous extract	17.41 nm (XRD crystallite size); 19.38 ± 2.14 nm (TEM); 5-35 nm size range; 325.6 nm hydrodynamic diameter (DLS)	Hexagonal (wurtzite crystal structure)	UV-Visible spectroscopy, FTIR, XRD, TEM, DLS, Zeta potential (−4.72 mV), EDX
(Yassin et al., 2025a)	Saudi Arabia	*C. albicans* (ATCC 90028), *C. glabrata* (ATCC 90876), *C. parapsilosis* (ATCC 22019), *C. tropicalis* (ATCC 13803)	Plant-mediated synthesis	*Trigonella foenum-graecum* (Fenugreek) aqueous seed extract	27.92 nm (TEM); 12.87 nm (XRD crystallite size); 169.6 nm (DLS hydrodynamic size)	Hexagonal (wurtzite)	UV–Vis, TEM, EDX, FTIR, XRD, DLS (Zetasizer), Zeta potential, SEM
(Yassin et al., 2025b)	Saudi Arabia	Drug-resistant *C. albicans* and *C. tropicalis*	Green synthesis	*Lepidium sativum* (garden cress) seed extract	Average diameter: 27.9 nm (TEM); crystallite size: 12.9 nm (XRD)	Hexagonal	UV-Visible spectroscopy, FTIR, XRD, TEM, DLS, Zeta potential (−18.3 mV), EDX

**Figure 1:**
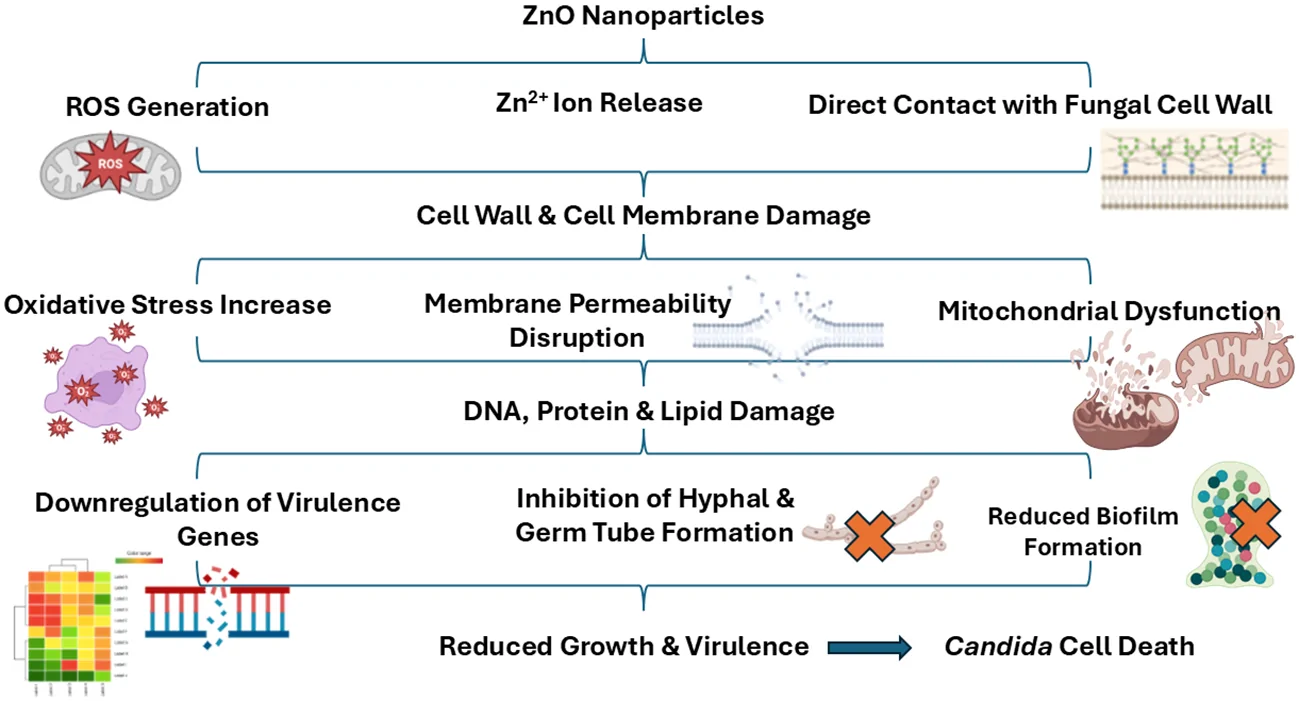
Proposed mechanisms of antifungal activity of zinc oxide nanoparticles against *Candida* species.

Membrane disruption is another thoroughly studied mechanism. Numerous studies have demonstrated that ZnO NPs induce structural alterations in the fungal cell wall and plasma membrane, including pore formation, membrane rupture, intracellular component leakage, and loss of cellular integrity (Reda and Fayed, 2023; Shoeb et al., 2013; Yassin et al., 2025a). Additionally, the dissolution of ZnO NPs yields Zn²^+^ ions, which may further enhance their increase antifungal activity by disrupting upsetting intracellular enzyme systems and metabolic pathways (Djearamane et al., 2022; Mohammed et al., 2024) (**Figure 1**).

**Figure 2:**
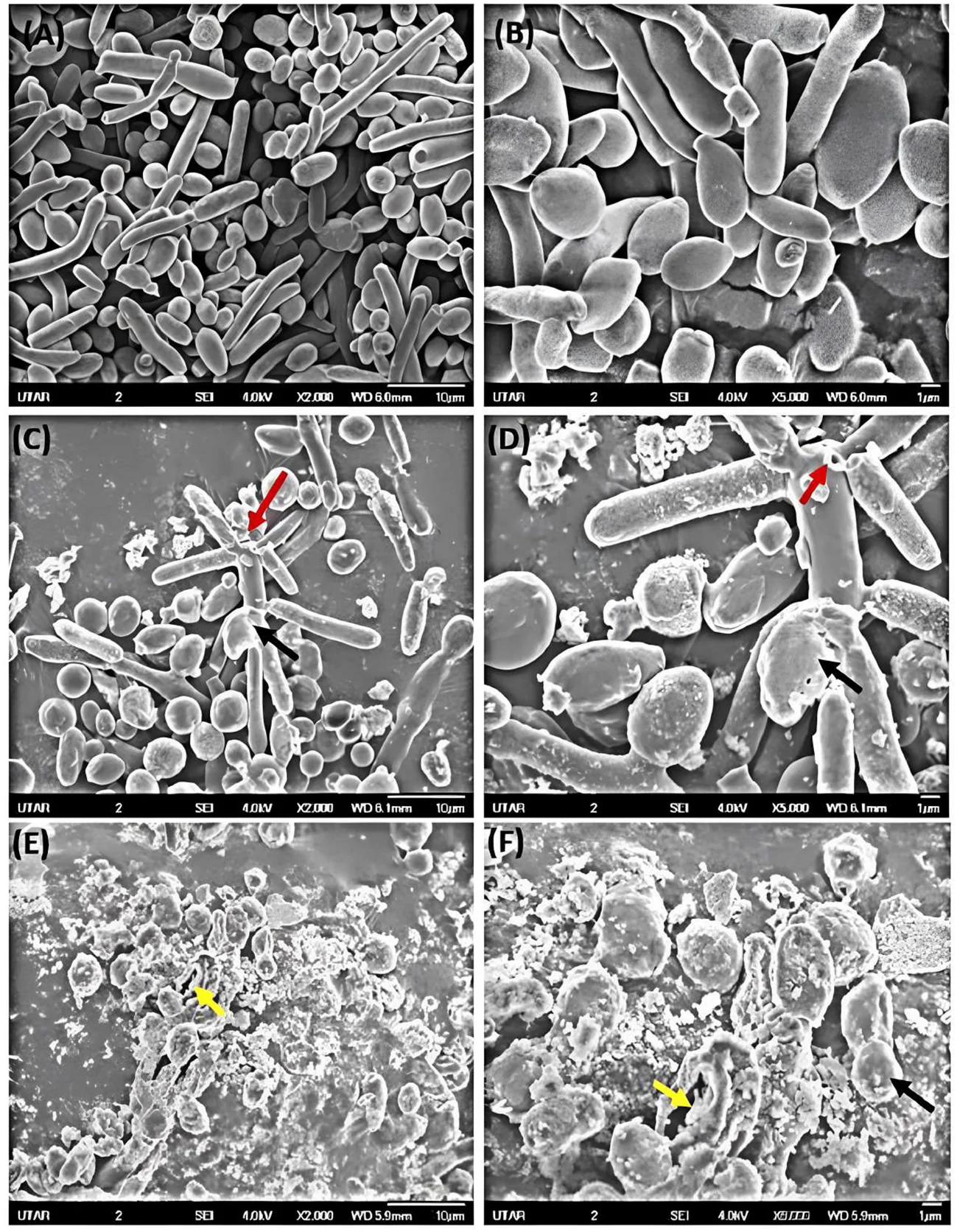
SEM images of *C. albicans* without ZnO NPs treatment (negative control) **(A, B)**; treatment with 80 µg/mL of ZnO NPs showed breakage of hyphae (red arrow) and pitting of yeast surface (black arrow) **(C, D)**; treatment with 160 μg/mL of ZnO NPs showed invagination of yeast cell surface (black arrow) and rupture of the cell membrane (yellow arrow) **(E, F)** after 24 h incubation. Adapted from Djearamane et al. (2022).

Beyond direct fungicidal activity, ZnO NPs also inhibit important virulence determinants of *Candida*. ZnO NPs may reduce fungal pathogenicity in addition to inhibiting fungal growth, according to several studies that showed decreased biofilm formation, inhibition of hyphal development, and downregulation of virulence-associated genes like *HWP1*, *ALS1*, *ALS3*, *ERG11*, *SAP*, and *RAS1* (Abedzadeh et al., 2022; Aslani et al., 2018; Fayed et al., 2025; Hosseini et al., 2020; Joshi et al., 2022). However, these molecular studies remain limited, as most studies used quantitative PCR to assess only one or two target genes, whereas thorough transcriptome, proteomic, and metabolomic analyses are nearly largely lacking. **Figure 2** summarizes the morphological alterations induced by ZnO NPs in *C. albicans*, as reported by Djearamane et al. (2022). SEM showed that whereas treatment with ZnO NPs resulted in significant structural damage, untreated *C. albicans* cells retained a smooth, intact cell surface and typical yeast shape. At higher nanoparticle concentrations (80 and 160 µg/mL), the treated cells showed hyphal breakdown, surface pitting, membrane invagination, and cell membrane rupture. These findings imply that ZnO NPs cause permanent cellular damage and loss of viability by compromising the structural integrity of the fungal cell wall and plasma membrane. Proteins, polysaccharides, phospholipids, and other cell wall-associated functional groups were shown to be involved in nanoparticle binding by FTIR analysis. Concurrently, EDX verified that treated fungal cells had zinc on their surface. The authors hypothesized that the antifungal activity of ZnO NPs is mediated by direct nanoparticle adhesion to the fungal surface, disruption of membrane integrity, induction of oxidative stress, and subsequent leakage, which ultimately leads to fungal cell death, based on these complementary analyses. These results offer crucial mechanistic evidence supporting the development as potential nanomaterials for the treatment of *Candida* infections (Djearamane et al., 2022).

The main conclusion is that research on the synthesis of nanoparticles has progressed faster than mechanistic studies. The majority of papers concentrated on nanoparticle synthesis, physicochemical characterization, and antifungal susceptibility testing; in-depth molecular investigations were rarely performed. This limitation is probably caused by the high expense of advanced analytical techniques, restricted access to omics platforms, and the transdisciplinary expertise needed for mechanistic research. Furthermore, it is more challenging to compare studies directly and reduces the repeatability of mechanistic results due to variations in fungal strains, surface chemistry, particle size, nanoparticle manufacturing methods, and experimental methodologies.

Even though other pathways like mitochondrial dysfunction, apoptosis-like cell death, intracellular signaling, and global metabolic regulation have not received enough attention, the evidence currently available strongly supports ROS generation and membrane disruption as the primary antifungal mechanisms of ZnO NPs. To further understand the intricate relationships between ZnO NPs and *Candida* cells, future research should incorporate transcriptomics, proteomics, metabolomics, sophisticated live-cell imaging, and standardized mechanistic experiments. In addition to improving our knowledge of nanoparticle-mediated antifungal action, these studies will make it easier to build safer and more potent nanotherapeutics.

## 5 ZnO NPs synergistic effect with traditional antifungal medications

Due to the increasing prevalence of multidrug-resistant *Candida* species and the toxicity of long-term antifungal treatment, combination approaches using ZnO NPs and conventional antifungal drugs have drawn attention. Few studies have examined ZnO NPs’ synergistic interactions with currently available antifungal drugs, despite the fact that they have shown strong intrinsic antifungal activity. Fluconazole was the medication that was most thoroughly studied in conjunction with ZnO NPs among the papers included in this study. Green-synthesized ZnO NPs greatly increased the antifungal activity of fluconazole against a variety of *Candida* species, especially *C. glabrata* and *C. parapsilosis*. Additionally, moderate synergistic effects against *C. albicans* and *C. tropicalis* were observed (Yassin et al., 2025a). Amphotericin B-conjugated ZnO NPs showed superior antifungal activity compared to unconjugated nanoparticles, according to Mathew et al. (2025), indicating that ZnO NPs may also be helpful drug delivery vehicles (Mathew et al., 2025). Additionally, ZnO-based nanocomposites containing vancomycin, silver, or chitosan showed enhanced antifungal activity, suggesting that surface modification of nanoparticles may further improve therapeutic efficacy (Abou-Dobara et al., 2025; Dananjaya et al., 2018; Das et al., 2016).

The enhanced effectiveness of these combinations is primarily due to ZnO NPs’ multifaceted mechanisms of action, which includes disruption of fungal membrane integrity, increased intracellular drug penetration, ROS-mediated oxidative stress, inhibition of biofilm formation, and interference with virulence-associated pathways (Aslani et al., 2018; Chand et al., 2024; Sharma and Ghose, 2015; Shoeb et al., 2013). These complementary processes may reduce dose-dependent toxicity, lower the effective dose of antifungal drugs, delay the development of resistance, and improving the management of multidrug-resistant and biofilm-associated *Candida* infections. However, despite these positive results, there are still strikingly few studies investigating synergistic interactions. Most of the experiments focused largely on nanoparticle manufacturing and antifungal screening rather than on combination therapy. Only a limited number of studies have confirmed the synergy using fractional inhibitory concentration index (FICI) analysis or standardized checkerboard experiments. Direct comparison between research is also challenging due to variations in fungal strains, physicochemical properties, susceptibility testing procedures, and nanoparticle manufacturing techniques. ZnO NPs may be used as adjunctive agents to increase the efficacy of conventional antifungal drugs, according to the information currently available; nevertheless, the database is still insufficient for clinical translation. Future studies should carefully assess combinations across all main antifungal classes, such as azoles, polyenes, echinocandins, and flucytosine, using standardized *in vitro* and *in vivo* models. Thorough mechanistic research and well planned animal experiments are also required to determine if nanoparticle-mediated synergy can lower toxicity, enhance treatment results, and potentially delay the emergence of antifungal resistance in therapeutically relevant settings. To establish ZnO NP-based combination treatment as a workable approach for treating drug-resistant candidiasis, such studies will be crucial.

## 6 Toxicity and safety considerations of ZnO NPs

Despite the promising antifungal activity of ZnO NPs, their therapeutic application largely hinges on establishing a safety profile acceptable to humans and the environment. The majority of the research in this review concentrated on antifungal activity without assessing biosafety; just a small portion evaluated cytotoxicity. At concentrations effective against *Candida*, green-synthesized ZnO NPs generally showed limited toxicity toward mammalian cells, according to the currently available evidence. Rare-earth doping has been shown to modify the antimicrobial and biocompatibility characteristics of ZnO nanostructures. Gd-doped ZnO nanorods exhibited selective antibacterial activity against *S. aureus* and relatively low toxicity in *Caenorhabditis elegans* and *Danio rerio*, while La doping enhanced the magnetic properties of ZnO and maintained narrow-spectrum antibacterial activity with low toxicity at 100 µg/mL. These findings suggest that dopant-induced modification of ZnO, potentially through changes in defect states such as oxygen vacancies, may be a promising strategy for enhancing the biological performance of ZnO nanomaterials; however, their specific antifungal efficacy against *Candida* species remains insufficiently explored (Satpathy et al., 2021a, b). Yassin et al. (2025a) found a high IC_50_ value against normal WI-38 fibroblast relative to the antifungal MIC, indicating a favorable therapeutic window. Similarly, Souza et al. (2020) observed excellent hemocompatibility with little hemolytic activity, while Aslani et al. (2018) showed minimal toxicity of chitosan-coated ZnO NPs against HepG2 cells.

One noteworthy finding of this study is that, rather than using primary human cells, most cytotoxicity studies employed immortalized or cancer-derived cell lines, such as HepG2. The physiological responses of healthy tissues are not accurately reflected in cancer cell lines, despite their widespread use as low-cost, reproducible, and easy-to-maintain models. As a result, the clinical safety of ZnO NPs may be either overestimated or underestimated based on toxicity data derived from transformed cells. Furthermore, very few studies have examined the effects of nanoparticle properties on toxicity, despite growing evidence that cytotoxicity is highly dependent on dose, particle size, shape, surface charge, coating materials, and Zn²^+^ ion release (Sharma and Ghose, 2015; Shoeb et al., 2013). Green synthesis and surface functionalization have generally been associated with improved biocompatibility, suggesting that nanoparticle design is essential to achieving a balance between host safety and antifungal activity.

Another crucial but frequently overlooked facet of ZnO NPs studies is environmental safety. None of the studies included in this review comprehensively evaluated the environmental fate, persistence, or ecotoxicological impact of ZnO NPs following biomedical application. The increasing use of nanoparticles in pharmaceutical formulations, coatings, and medical devices raises concerns regarding their accumulation in aquatic environments and their effects on beneficial bacteria. While there is evidence of ZnO NPs’ short-term biocompatibility, long-term environmental and human safety remain poorly understood, and only a small portion of research has successfully addressed toxicity. Future research should focus on standardized toxicity evaluation using normal human cell lines, repeated-dose animal studies, pharmacokinetic and biodistribution assessments, and environmental risk assessment to facilitate the safe clinical translation of ZnO NP-based antifungal medications.

## 7 Limitations, challenges, and future directions

**Figure 3:**
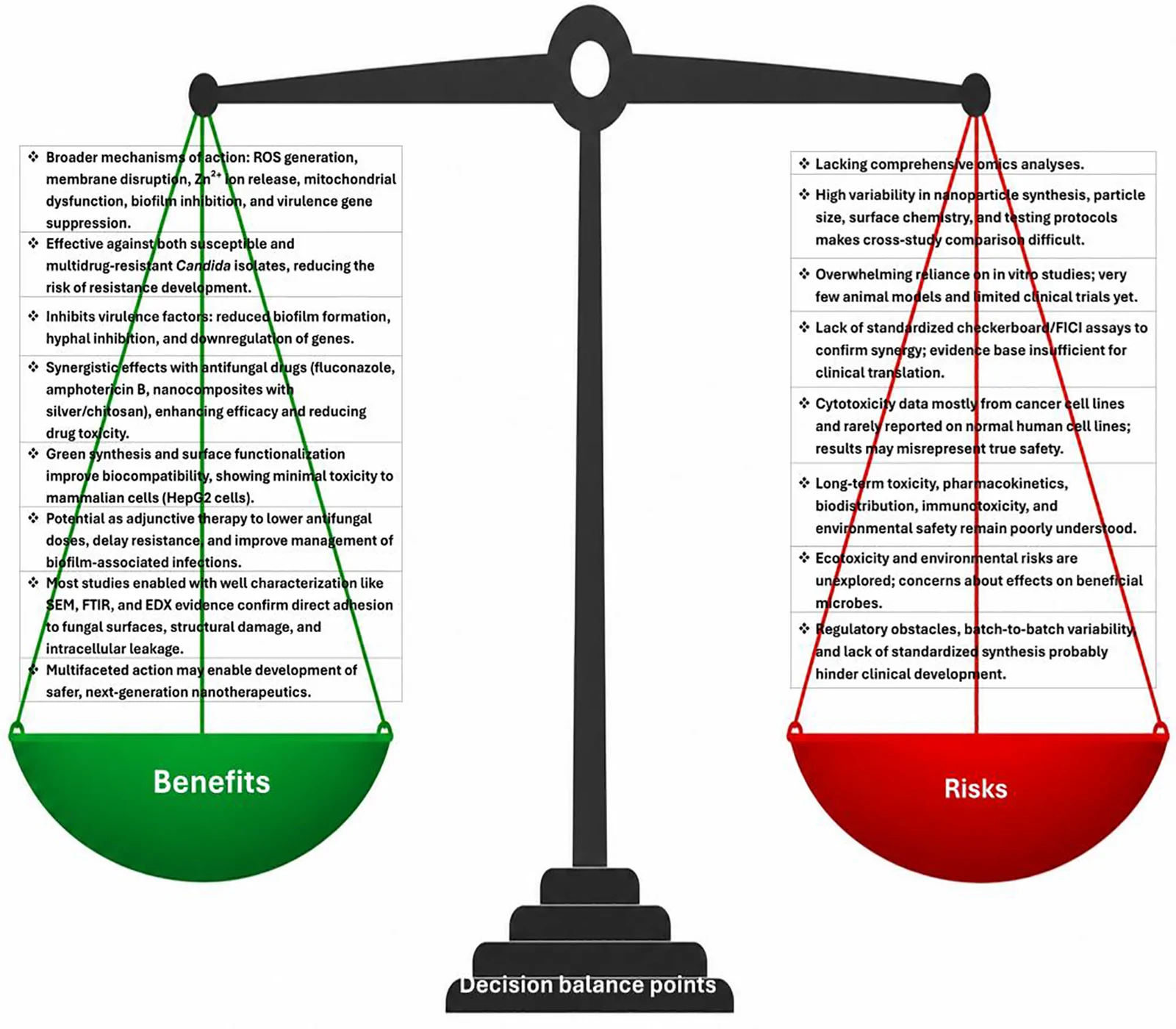
Risk-benefit assessment of zinc oxide nanoparticles as antifungal agents against *Candida* species.

One of the main issues noted in this study is the considerable variance in nanoparticle synthesis, physicochemical characterization, and biological evaluation across studies. Zn²^+^ ion release, surface charge, crystallinity, particle size, and morphology, vary considerably among different studies (Albuquerque et al., 2023; Mathew et al., 2025; Shoeb et al., 2013; Souza et al., 2020; Yassin et al., 2025a). Because these physicochemical characteristics directly affect antifungal activity, biofilm inhibition, ROS production, and cytotoxicity, it remains difficult to compare results across studies. Therefore, standardized characterization of critical parameters, including zeta potential, colloidal stability, hydrodynamic size, and dissolution kinetics, is essential. The main therapeutic advantages current hazards, and limitations of ZnO NPs for the treatment of candidiasis are shown in **Figure 3**. Their diverse antifungal mechanisms, antivirulence and antibiofilm activities, synergistic potential with conventional antifungals, and favorable biocompatibility are summarized in the left panel. In contrast, the right panel highlights the main obstacles, such as the lack of *in vivo* and clinical evidence, variability in the synthesis and characterization of nanoparticles, unresolved toxicity and environmental concerns, and obstacles to standardization and clinical translation.

Although most of the heterogeneity is caused by differences in experimental design, inoculum size, incubation conditions, biofilm tests, and endpoint determination, the lack of established protocols for assessing antifungal susceptibility is another significant disadvantage. Similarly, there are still very few mechanistic studies, most of which concentrate on ROS generation and membrane disruption and mostly rely on indirect data from electron microscopy or fluorescence-based tests. There are very few comprehensive transcriptomic, proteomic, metabolomic, and functional genomic studies investigating the molecular responses of *Candida* to ZnO NP exposure. As a result, the precise molecular mechanisms underlying ZnO NP-mediated antifungal activity of these interactions remain poorly understood.

The greatest obstacle to clinical application is the overreliance on *in vitro* studies. There were no clinical trials assessing ZnO NPs for the treatment of candidiasis, and only a small number of investigations advanced to animal models. In a similar vein, some research has been examined environmental safety and reproductive toxicity. These gaps are still delaying the clinical development of ZnO NPs-based antifungal medications, regulatory barriers, batch-to-batch variability, and inconsistent production methods. Therefore, the top priorities for future research should be standardized synthesis and characterization protocols, clinically relevant toxicity assessment using animal models and normal human cell lines, comprehensive molecular investigations using omics technologies, and systematic evaluation of combination therapies with conventional antifungal agents. Close interdisciplinary collaboration will be essential to overcoming these challenges and enabling the safe and effective clinical translation of ZnO NPs as next-generation antifungal therapies.

## 8 Conclusion

Research indicates that ZnO NPs are environmentally friendly, broad-spectrum antifungal options for treating *Candida* infections. Based on the studies, different mechanisms of action have been proposed for the antifungal activity of ZnO NPs against both sensitive and multidrug-resistant *Candida* strains: increased oxidative stress, disrupted cell membranes, Zn^2+^ release from ZnO NPs, and inhibition of biofilm formation and *Candida* virulence factors. These ZnO NPs offer additional advantages over traditional antifungal drugs: their broad-spectrum activity and the ability to be produced via green synthesis make them much more biocompatible than synthetic drugs and enable the development of combined therapies with traditional antifungal medications to improve efficacy and prevent resistance. Although ZnO NPs are effective antifungal agents against *Candida* species, clinical evidence supporting the routine clinical application requires further validation. Future studies should focus on standardized *in vitro* and *in vivo* methods, comprehensive toxicological and pharmacokinetic evaluations, and well-designed planned clinical trials to determine their safety, efficacy, and optimal therapeutic use in the prevention and treatment of candidiasis.
