# Supplementary material for: Zinc oxide nanoparticles against Candida: mechanistic pathways, synergistic potentials, and biocompatibility challenges in the era of antimicrobial resistance
Source: Front Antibiot. 2026 Sep 11;5:1933663. doi: 10.3389/frabi.2026.1933663 (PMC13613811; doi:10.3389/frabi.2026.1933663)
Supplement: Supplementary file 1 [file Table1.docx]

**Zinc oxide nanoparticles against *Candida*: Mechanistic pathways, synergistic potentials, and biocompatibility challenges in the era of antimicrobial resistance**

**Binaya Krushna Sahu^1^, Tapan Kumar Mohanta^2^, Mahesh Chandra Sahu^3^ and Sujogya Kumar Panda^1, *^**

^1^Centre for Biotechnology, Siksha ‘O’ Anusandhan (Deemed to be University), Kalinganagar, Bhubaneswar-751003, Odisha, India

^2^Dept. of Genetic Research, Institute for Research and Medical Consultations, Imam Abdulrahman Bin Faisal University, Dammam, 34221, Saudi Arabia.

^3^Division of Microbiology, ICMR-Regional Medical Research Centre, Chandrasekharpur, Bhubaneswar-751023, Odisha, India

**Corresponding Author*** Dr. Sujogya Kumar Panda, Email: [sujogyapanda@soa.ac.in](mailto:sujogyapanda@soa.ac.in)

**Table S1.** Antifungal activity of zinc oxide nanoparticles against *Candida* species, including antifungal assays, MIC/MFC values, biofilm inhibition, mechanisms of action, synergistic effects, cytotoxicity, and major findings.

| **Reference** | **Antifungal Assay** | **MIC/MFC Values** | **Biofilm Inhibition Results** | **Mechanism(s) of Action** | **Synergy with Antifungal Drugs** | **Cytotoxicity/Biocompatibility Findings** | **Major Conclusion** |
| --- | --- | --- | --- | --- | --- | --- | --- |
| (Abedzadeh et al., 2022) | CLSI disk diffusion assay, broth microdilution MIC assay, Real-time PCR (ERG11, CDR2, ALS1, HWP1 gene expression) | ZnO-NPs: MIC50 = 19.9 µg/mL, MIC90 = 44.7 µg/mL; Fluconazole: MIC50 = 7 µg/mL, MIC90 = 14.1 µg/mL | No direct biofilm assay performed. However, ZnO-NPs + fluconazole significantly downregulated biofilm-associated genes (ALS1 decrease 5.5-fold, HWP1 decrease 3-fold) indicating inhibition of biofilm formation | ZnO-NPs downregulated ERG11, CDR2, ALS1, and HWP1 gene expression, suppressed efflux pumps, reduced adhesion and virulence gene expression, and enhanced antifungal susceptibility. | Yes. A combination of ZnO-NPs and fluconazole exhibited significant synergistic activity, markedly reducing the expression of ERG11, CDR2, ALS1, and HWP1 compared with either treatment alone. | - | ZnO-NPs combined with fluconazole exhibited superior antifungal activity against resistant *C. albicans* by suppressing resistance and virulence genes, suggesting their potential as an adjunct therapy for vulvovaginal candidiasis |
| (Abou-Dobara et al., 2025) | Agar well diffusion assay, Broth dilution MIC, Minimum Fungicidal Concentration (MFC), TEM ultrastructural analysis | MIC = 5 µg/mL; MFC = 5 µg/mL | - | ZnO/CS/VA nanocomposite released Zn²⁺ ions. It generated ROS, causing membrane disruption, cytoplasmic leakage, separation of the cell wall and membrane, vacuole formation, protein denaturation, DNA damage, and fungal cell death. Smaller nanoparticles enhanced cellular penetration and ROS production. | Yes. Vancomycin incorporated into the ZnO/chitosan nanocomposite enhanced anti*Candida*l activity through a synergistic multifunctional effect, producing a larger inhibition zone than ZnO NPs or fluconazole alone. | - | Green-synthesized ZnO/chitosan/vancomycin nanocomposite exhibited potent anti*Candida*l activity against *C. albicans*, showing a 31 mm inhibition zone and MIC/MFC of 5 µg/mL, outperforming fluconazole and demonstrating promise as a future antifungal nanotherapeutic |
| (Ahmadpour Kermani et al., 2021) | CLSI M27-ED4 broth microdilution (MIC/MFC), agar well diffusion, MTT cytotoxicity assay | ZnO: MIC 64–128 μg/mL; MFC 256–512 μg/mL. TiO₂: MIC 128–256 μg/mL; MFC 256–512 μg/mL. AmB: MIC 8–16 μg/mL; MFC 16–32 μg/mL | - | - | - | MTT assay on J774 mouse macrophages. CC₅₀: TiO₂ = 862.1 μg/mL; ZnO = 706.2 μg/mL; AmB = 70.19 μg/mL. Nanoparticles showed significantly lower cytotoxicity than Amphotericin B. | ZnO and TiO₂ nanoparticles inhibited all tested *Candida* species. ZnO exhibited stronger antifungal activity than TiO₂, but both were less potent than Amphotericin B. Both nanoparticles demonstrated markedly lower cytotoxicity, indicating potential as safer antifungal agents. |
| (Albuquerque et al., 2023) | Disk diffusion assay (CLSI guidelines); Zone of inhibition (ZOI) measured | - | - | Antifungal activity attributed to ZnO-induced ROS generation, Zn²⁺ ion release, disruption of fungal cell membrane integrity, and reduction/oxidation reactions during biosynthesis | - | The literature suggests that ZnO-NPs have minimal effects on mammalian cells, but this study did not conduct cytotoxicity or biocompatibility testing. | Both chemically and biologically synthesized ZnO-NPs inhibited *C. albicans*. Optimal concentrations were 7 wt% for chemically synthesized ZnO-NPs and 9 wt% for biologically synthesized ZnO-NPs. Chemically synthesized ZnO-NPs exhibited greater antifungal efficacy, whereas biologically synthesized ZnO-NPs offer an environmentally friendly alternative. |
| (Alves et al., 2018) | Colony-forming unit (CFU) assay, biofilm colonization assay, SEM imaging, viability assay | MIC/MFC not reported | ZnO rod-like coating significantly inhibited biofilm formation. For *C. albicans*, biofilm maturation was impaired despite surface colonization. For *C. parapsilosis*, both surface colonization and biofilm formation were significantly reduced. | ZnO coating altered surface properties, reducing cell-to-cell and cell-to-surface adhesion, thereby impairing biofilm development. The local release of Zn²⁺ ions and/or ROS has been proposed to reduce adhesion. The coating also promoted precipitation of calcium phosphate (CaP)-derived compounds while maintaining antibiofilm activity. | - | The precipitation of CaP-derived compounds on the ZnO coating suggests good biocompatibility and potential suitability for bone-related biomedical applications, even in the presence of *Candida* infection. | Microstructured ZnO rod-like coatings effectively inhibited biofilm formation by *C. albicans* and *C. parapsilosis*. Although fungal cells could eventually disassemble the coating during prolonged colonization, the coating retained antibiofilm activity and supported CaP deposition, indicating promise for antimicrobial bone implant applications |
| (Anitha et al., 2023) | Broth microdilution (MIC), Crystal Violet assay, XTT assay, Growth curve analysis | MIC = 48 µg/mL; MFC = Not reported | Up to 78% inhibition (Crystal Violet assay) and 63% inhibition (XTT assay) at sub-MIC concentrations (4, 8, and 16 µg/mL) | Inhibited fungal growth and biofilm formation; reduced adherence and virulence without significant cytostatic effects at sub-MIC concentrations | - | - | Green-synthesized flower-shaped ZnO NPs from cashew apple juice showed potent antifungal and antibiofilm activity against fluconazole-resistant *C. glabrata* and have potential as an alternative or adjunctive therapy for biofilm-associated candidiasis |
| (Aslani et al., 2018) | CLSI broth microdilution (MIC), MTT biofilm assay, qRT-PCR for *Hwp1* gene expression, ROS assay, cytotoxicity (MTT) | MIC = 32 µg/mL; MFC not reported | ZnO NPs significantly reduced *C. albicans* biofilm formation at the MIC concentration (P = 0.006) and markedly decreased *Hwp1* gene expression in both standard and clinical isolates (P = 0.001) | ZnO NPs increased intracellular ROS production in a dose-dependent manner, downregulated the biofilm-associated *Hwp1* gene, inhibited biofilm formation, and consequently reduced *Candida* pathogenicity. | - | Minimal cytotoxicity toward HepG2 human hepatocyte cells at 32–128 µg/mL; no significant toxicity observed. | Novel chitosan-linoleic acid-coated ZnO NPs effectively inhibited *C. albicans* biofilm formation by suppressing *Hwp1* gene expression while exhibiting minimal cytotoxicity, indicating their potential as a safe antifungal nanotherapeutic against biofilm-associated candidiasis |
| (Dananjaya et al., 2018) | Broth microdilution assay for MIC determination, FESEM analysis of fungal morphology, cytotoxicity assay using HEp-2 cells | MIC: ZnO NPs = 200 µg/mL; ZnO–chitosan nanocomposites = 75 µg/mL; MFC not reported | - | ZnO–chitosan nanocomposites damaged the fungal cell membrane, altered cell morphology, and enhanced antifungal activity through the combined effects of ZnO nanoparticles and chitosan. Chitosan improved nanoparticle interactions with fungal cells, resulting in a synergistic antifungal effect. | - | ZnO-chitosan nanocomposites exhibited significantly lower cytotoxicity toward HEp-2 human epithelial cells than ZnO nanoparticles, demonstrating good cytocompatibility and potential for biomedical applications. | ZnO-chitosan nanocomposites showed superior antifungal activity against *Candida albicans* compared with ZnO nanoparticles alone, with a lower MIC (75 µg/mL), enhanced fungal cell membrane damage, and improved biocompatibility, indicating their potential as promising antifungal nanomaterials for biomedical applications. |
| (Das et al., 2016) | Broth microdilution assay, Growth kinetics, Colony-forming unit (CFU) assay, ROS detection, Lipid peroxidation assay, DNA fragmentation, SEM/TEM imaging, Live–dead staining | MIC = 250 µg/mL; MFC not reported | Not directly evaluated; however, Ag@ZnO nanocomposites caused extensive membrane damage, cellular leakage, and destruction of fungal cells, which may prevent biofilm establishment. | Ag@ZnO nanocomposites generated excessive reactive oxygen species (ROS), induced lipid peroxidation, disrupted cell membrane integrity, caused DNA fragmentation, leakage of intracellular components, mitochondrial dysfunction, and ultimately fungal cell death through oxidative stress-mediated apoptosis. | - | Cytotoxicity was evaluated using A431 human epidermoid carcinoma cells. Ag@ZnO nanocomposites exhibited significantly lower cytotoxicity than Ag nanoparticles while maintaining high antifungal activity, indicating improved biocompatibility. | Ag@ZnO core–shell nanocomposites demonstrated significantly greater antifungal activity than Ag or ZnO nanoparticles alone against *Candida krusei*. The enhanced efficacy was attributed to synergistic ROS-mediated oxidative damage and membrane disruption, suggesting Ag@ZnO nanocomposites as promising antifungal nanomaterials for biomedical applications. |
| (Djearamane et al., 2022) | Growth inhibition by turbidity (OD600), colony count, INT (iodonitrotetrazolium chloride) assay, SEM, SEM-EDX, and FTIR | MIC = 80 µg/mL; EC_50_ = 35.59 µg/mL MFC not reported | - | ZnO NPs accumulated on the fungal surface, interacted with cell-wall functional groups (alcohol, amide A, methyl, alkynes, amide I & II, phosphate), caused hyphal damage, pitting of the cell wall, membrane invagination, and membrane rupture, ultimately compromising membrane integrity and leading to fungal cell death. A dose-dependent inhibitory effect was observed | - | - | ZnO NPs exhibited significant dose-dependent antifungal activity against *C. albicans*. Surface adsorption of nanoparticles induced structural damage to fungal cells and inhibited growth, highlighting ZnO NPs as promising antifungal nanomaterials for future biomedical applications |
| (Fayed et al., 2025) | MIC₅₀ determination, XTT biofilm adhesion assay, gene-expression analysis (RT-qPCR) for resistance and adhesion genes | MIC₅₀: ZnO-NP-C1 = 61.9 ± 3.3 µg/mL; ZnO-NP-C2 = 151 ± 7.83 µg/mL; ZnO-NP-B = 1000 µg/mL (1 mg/mL). MFC not reported. | ZnO-NP-C1 showed the greatest antibiofilm activity, reducing *C. auris* adhesion by 67.9 ± 2.35% at 150 µg/mL. ZnO-NP-C2 inhibited adhesion by 51.6 ± 1.26%, whereas biologically synthesized ZnO-NP-B showed negligible inhibition. ZnO-NP-C1 significantly downregulated the adhesion genes *ALS5*, *IFF4*, and *CSA1*, preventing early biofilm formation. | Chemically synthesized ZnO nanoparticles induced oxidative stress, disrupted fungal membranes and cell walls, and prevented adaptive resistance by downregulating resistance genes (*CDR1, MDR1, ERG2, ERG11, CHS1,* and *FKS1*). ZnO-NP-C1 also suppressed adhesion-related genes (*ALS5, IFF4,* and *CSA1*), thereby inhibiting biofilm initiation. In contrast, biologically synthesized ZnO-NP-B upregulated several resistance genes, suggesting possible induction of adaptive resistance. | - | - | Chemically synthesized ZnO nanoparticles, particularly ZnO-NP-C1, exhibited significantly greater antifungal and antibiofilm activity than biologically synthesized ZnO nanoparticles against multidrug-resistant *C. auris*. Their smaller particle size and favorable physicochemical properties enabled effective inhibition of adhesion, suppression of resistance and virulence genes, and prevention of biofilm formation, highlighting their potential as antifungal coatings for medical devices and future therapeutic applications. |
| (Fozouni and Tahaei, 2023) | CLSI M27 broth microdilution assay for fluconazole susceptibility; broth dilution assay for ZnO NP antifungal activity; colony-forming unit (CFU) determination; MIC and MFC determination. | ZnO NPs: MIC50 = 7.43 μg/mL (*C. albicans*), 14.86 μg/mL (*C. famata*); MIC90 = 29.75 μg/mL (*C. albicans*), 59.5 μg/mL (*C. famata*); MFC = 119 μg/mL for both species. Fluconazole: MIC50 = 8 μg/mL (*C. albicans*), 16 μg/mL (*C. famata*); MIC90 = 128 μg/mL (*C. albicans*), 256 μg/mL (*C. famata*); MFC = 256 μg/mL and 512 μg/mL, respectively. | - | ZnO NPs exhibited dose-dependent fungicidal activity. Increasing nanoparticle concentration enhanced inhibition of fluconazole-resistant isolates, with complete killing at 119 μg/mL. The authors discuss antimicrobial activity in relation to nanoparticle size and shape, but did not experimentally investigate molecular mechanisms. | -. | - | ZnO NPs demonstrated potent in vitro antifungal activity against fluconazole-resistant *Candida* isolates from calves with diarrhea. All resistant isolates were eliminated at 119 μg/mL, indicating that ZnO NPs could serve as a promising alternative therapeutic strategy for gastrointestinal candidiasis in livestock, warranting future in vivo investigation. |
| (Hameed et al., 2015) | Agar disc diffusion assay, growth curve (OD₆₆₀), Minimum Fungicidal Concentration (MFC), histidine (ROS scavenger) assay, FESEM, zeta potential analysis. | MFC: 2000 μg/mL for Mg-doped ZnO, producing complete inhibition of *C. albicans*. MIC not reported. | - | Mg-doped ZnO exhibited the strongest antifungal activity due to enhanced ROS generation, increased oxygen vacancies, Zn²⁺ ion release, electrostatic attraction to negatively charged fungal cells, membrane disruption, and accumulation of nanoparticles on the cell surface. Histidine reduced antifungal activity, confirming the role of ROS. | - | - | Mg²⁺-doped ZnO nanoparticles demonstrated significantly greater antifungal activity against *Candida albicans* than pure ZnO or Ca²⁺-, Sr²⁺-, and Ba²⁺-doped ZnO nanoparticles. Their superior activity was attributed to enhanced photocatalytic activity, increased ROS production, smaller crystallite size, oxygen vacancies, and stronger electrostatic interaction with fungal cells. |
| (Hosseini et al., 2018) | Broth microdilution MIC assay, catheter adhesion assay, biofilm formation assay on urinary catheter, Crystal Violet biomass assay, CFU assay, SEM visualization of biofilms | MIC: 28 ± 1.2 μg/mL (fluconazole-susceptible isolates); 47 ± 2.8 μg/mL (fluconazole-resistant isolates). MFC not reported. | ZnO NPs completely inhibited biofilm formation in 80% of susceptible isolates at 29 μg/mL and 100% of fluconazole-resistant isolates at 50 μg/mL. Sub-MIC concentrations reduced biofilm formation in 58.3% of susceptible isolates and 60% of resistant isolates. Treatment of preformed biofilms destroyed >90% of biofilm biomass after 24 h and reduced CFUs by 15-fold (susceptible) and 100-fold (resistant) isolates. | The mechanism was not directly investigated. The authors suggest that ZnO nanoparticles inhibit fungal adhesion and biofilm development, likely through ROS generation, membrane disruption, hydrogen peroxide production, and Zn²⁺ ion release, consistent with previous reports. | - | - | ZnO nanoparticles effectively inhibited adhesion, biofilm formation, and preformed biofilms of Candida albicans isolates from urinary catheters, both fluconazole-susceptible and fluconazole-resistant. Their strong antibiofilm activity suggests potential application as an alternative strategy for preventing and treating catheter-associated candidiasis. |
| (Hosseini et al., 2019) | CLSI broth microdilution MIC assay, MTT biofilm assay, adhesion assay, Real-time PCR | MIC range: 0.02–296 µg/mL; MIC₅₀ = 8.2 µg/mL; MIC₉₀ = 17.76 µg/mL; MFC not reported | Sub-MIC concentrations significantly reduced initial adhesion (2 h), decreased biofilm metabolic activity, and downregulated ALS1 and ALS3 genes; however, mature 24-h biofilms were not completely inhibited. | ZnO nanoparticles generated reactive oxygen species (ROS), disrupted fungal membrane integrity, inhibited adhesion, and significantly downregulated the biofilm-associated genes ALS1 and ALS3, thereby reducing fungal virulence. | - | - | Chemically synthesized ZnO nanoparticles effectively inhibited the growth and initial adhesion of fluconazole-resistant *Candida albicans* isolates from UTIs. The authors suggested that ZnO nanoparticles could be used as coatings for medical devices to reduce fungal adhesion and catheter-associated candidiasis. |
| (Hosseini et al., 2020) | CLSI M44 disk diffusion assay, broth microdilution to determine sub-MIC concentrations, quantitative real-time PCR for SAP1, SAP2, and SAP3 gene expression. | Sub-MIC range of ZnO-NPs: 0.02–12 µg/mL (strain dependent); working sub-MIC ≈ 3 ± 1.7 µg/mL for gene-expression experiments. MIC and MFC values were not reported. | - | ZnO NPs significantly downregulated SAP1, SAP2, and SAP3 secreted aspartyl proteinase genes at sub-MIC concentrations, thereby reducing fungal virulence. Growth inhibition was also observed. | - | - | ZnO NPs inhibited the growth of fluconazole-resistant *C. albicans*. They significantly reduced SAP1–SAP3 gene expression at sub-inhibitory concentrations, indicating that ZnO NPs may reduce fungal virulence and represent a promising alternative therapeutic approach for vulvovaginal candidiasis. |
| (Jalal et al., 2018) | CLSI broth microdilution assay, growth inhibition assay, germ tube inhibition assay, phospholipase assay, proteinase assay, confocal laser scanning microscopy (CLSM) for biofilm analysis, SEM, and TEM | MIC = 0.25 mg/mL (250 µg/mL); MFC not reported | ZnO NPs inhibited biofilm formation by approximately 85% at 0.25 mg/mL. Germ tube formation was reduced by 86.4%, phospholipase activity by 58.8%, and proteinase secretion by up to 95.2%, indicating strong suppression of major virulence factors. | ZnO NPs adhered to and penetrated fungal cells, disrupted the cell wall and cytoplasmic membrane, induced structural deformation, and generated reactive oxygen species (ROS), leading to oxidative damage, inhibition of growth, and suppression of virulence factors, including germ tube formation, biofilm development, phospholipase, and proteinase secretion. | - | - | Green-synthesized ZnO nanoparticles exhibited potent dose-dependent anticandidal activity against clinical *Candida* isolates by inhibiting fungal growth, biofilm formation, germ tube development, and hydrolytic enzyme production while causing severe ultrastructural damage to fungal cells. The findings suggest that ZnO nanoparticles could be developed as antifungal coatings for medical devices to prevent catheter-associated candidiasis and other biofilm-related infections. |
| (Joshi et al., 2022) | Cell viability assay, MIC determination, morphogenesis inhibition assay, biofilm inhibition assay, CLSM, ROS assay, RT-PCR gene-expression analysis, in vitro cytotoxicity (3T3-L1 cells), in vivo rat toxicity study | MIC₅₀: L-ZnO = 465.8 µg/mL; FL-ZnO = 7.31 µg/mL; OFL-ZnO = 31.25 µg/mL. Morphogenesis inhibition MIC: 500, 31.2, and 62.5 µg/mL for L-ZnO, FL-ZnO, and OFL-ZnO, respectively. MFC not reported. | FL-ZnO nanoparticles showed the strongest antibiofilm activity, inhibiting >80% of biofilm formation at 31.25 µg/mL. Approximately 50% biofilm inhibition occurred at 3.25 µg/mL for FL-ZnO, compared with 262 µg/mL (L-ZnO) and 29.48 µg/mL (OFL-ZnO). Morphogenesis inhibition exceeded 90% at 31.2 µg/mL. | ZnO nanoparticles induced intracellular ROS generation, inhibited yeast-to-hyphae transition, suppressed morphogenesis, and modulated virulence-related genes by downregulating *phr1, phr2, efg1, hwp1, ras1, als3,* and *als4* while upregulating *bcy1, nrg1,* and *tup1*, leading to reduced virulence and biofilm formation. | - | FL-ZnO nanoparticles were biocompatible and non-toxic in vitro (3T3-L1 mouse fibroblast cells) and showed no significant toxicity in vivo in rat studies, indicating good biosafety. | Hierarchical FL-ZnO nanoparticles synthesized using fragmented lignin exhibited superior antifungal and anti-virulence activity compared with conventional ZnO nanoparticles. Their potent inhibition of growth, morphogenesis, biofilm formation, and virulence gene expression, together with excellent biocompatibility, highlights their potential as sustainable antifungal nanomaterials for controlling *Candida albicans* infections. |
| (Jothiprakasam et al., 2017) | XTT reduction assay and ATP bioluminescence (ATPase) assay for biofilm inhibition; comparison with fluconazole and EDTA | MIC/MFC values were not reported. ZnO NPs inhibited biofilms at 5.2 mg/mL (fluconazole-susceptible strain) and 5.42 mg/mL (fluconazole-resistant strain). EDTA required 8.6 and 10.8 mg/mL, respectively. | ZnO nanoparticles markedly inhibited biofilm formation on both fluconazole-susceptible and fluconazole-resistant *C. tropicalis*. Biofilm metabolic activity and cell viability were significantly reduced as measured by XTT and ATPase assays. ZnO NPs were more effective than EDTA at lower concentrations and effectively prevented biofilm development on catheter surfaces. | The antifungal activity was attributed to the generation of intracellular reactive oxygen species (ROS), oxidative damage to cellular lipids and coenzyme A, disruption of respiratory processes, and subsequent fungal cell death. | - | - | Egg albumin-mediated ZnO NPs effectively inhibited biofilm formation by both fluconazole-susceptible and fluconazole-resistant *Candida tropicalis*. ZnO nanoparticles showed greater antibiofilm efficacy than EDTA at lower concentrations and were proposed as promising coating materials for preventing catheter- and medical device-associated candidal biofilm infections. |
| (Katafa and Hamid, 2020) | Agar well diffusion assay (zone of inhibition) | MIC/MFC not reported; Zone of inhibition at 800 mg/mL: 11 mm (calcined ZnO NPs) and 24 mm (non-calcined ZnO NPs) | - | - | - | Toxicity observed in vivo: Histopathological examination of mice showed liver congestion and proliferation of megakaryocytes in the spleen after exposure to ZnO nanoparticles, indicating dose- and time-dependent toxicity | Chemically synthesized ZnO NPs exhibited concentration-dependent antifungal activity against *C. albicans*. Non-calcined ZnO NPs demonstrated superior antifungal efficacy compared with calcined nanoparticles because of their smaller particle size. However, ZnO NPs also produced dose-dependent toxic effects in mice, highlighting the need for careful safety evaluation |
| (Maniah, 2023) | Disk diffusion assay, broth microdilution (MIC/MFC), checkerboard synergy assay (IFA), SEM, MTT cytotoxicity assay | MIC = 100 µg/mL; MFC = 200 µg/mL (highest activity against *Candida tropicalis*) | - | ZnO nanoparticles exerted antifungal activity through the generation of reactive oxygen species (ROS), Zn²⁺ ion release, disruption of fungal cell membranes, oxidative stress, and interactions between phytochemical capping molecules (alcohols, phenols, amines, and aldehydes) and fungal cells, resulting in cell death. | Yes. The strongest synergistic activity was observed with clotrimazole against *Candida glabrata*, followed by *Candida auris*. Combination treatment significantly enhanced antifungal activity compared with either agent alone. | The MTT assay showed low cytotoxicity toward normal WI-38 human lung fibroblasts, with an IC₅₀ of 774.45 µg/mL, indicating excellent biocompatibility and biosafety for biomedical applications. | Green-synthesized *Laurus nobilis*-mediated ZnO nanoparticles exhibited potent broad-spectrum anticandidal activity against drug-resistant *Candida* pathogens and significantly enhanced the efficacy of clotrimazole. Their small particle size, favorable biocompatibility, and eco-friendly synthesis support their potential as safe nano-adjuvant antifungal agents for treating resistant candidal infections. |
| (Mathew et al., 2025) | Agar well diffusion (zone of inhibition), growth inhibition assay, and pH-dependent drug release study | MIC/MFC not reported; Zone of inhibition against *C. albicans*: 47 ± 0.49 mm (AmB@ZnONPs), 41 ± 0.58 mm (AmB), 37 ± 0.62 mm (ZnONPs) | - | Enhanced antifungal activity through ROS generation, Zn²⁺ ion release, phytochemical-mediated membrane disruption, and pH-sensitive sustained release of Amphotericin B. Drug conjugation involved electrostatic, hydrogen bonding, hydrophobic, van der Waals, coordination, and π–π interactions, improving stability and targeted delivery | ZnO NPs showed synergistic enhancement with Amphotericin B, producing a larger inhibition zone than either agent alone. | Improved biocompatibility with reduced hemolytic toxicity, enhanced aqueous solubility, improved bioavailability, controlled drug release, and lower expected systemic toxicity compared with conventional Amphotericin B | Green-synthesized *Mangifera indica* leaf-mediated ZnO NPs conjugated with Amphotericin B exhibited significantly enhanced anti*Candida*l activity, improved drug solubility and stability, pH-responsive sustained release, reduced toxicity, and represent a promising nanocarrier for targeted antifungal therapy against *C. albicans*. |
| (Metwally et al., 2022) | Disc diffusion assay, Broth dilution assay (MIC and MFC), MTT cytotoxicity assay | MIC = 0.25 mg/mL; MFC = 0.5 mg/mL (all tested *Candida* isolates) | - | Proposed mechanisms include cell membrane disruption; inhibition of ergosterol synthesis and cell wall formation; interference with the mitotic spindle and DNA transcription; and electrostatic interactions between positively charged ZnO NPs and negatively charged fungal cell walls, leading to cell death. | - | IC₅₀ = 230.12 ± 9.34 µg/mL against normal human lung fibroblast (MRC-5) cells, indicating acceptable biocompatibility at antifungal concentrations. | Green-synthesized lemon peel-mediated ZnO NPs exhibited significant anti*-Candida* activity against MDR *Candida* species, with inhibition zones up to 24.5 ± 0.5 mm, an MIC of 0.25 mg/mL, an MFC of 0.5 mg/mL, and acceptable cytocompatibility, demonstrating their potential as eco-friendly antifungal nanotherapeutics. |
| (Mohammed et al., 2024) | Agar well diffusion assay (zone of inhibition) | MIC/MFC not reported; Zone of inhibition: 18 mm (400 µg/mL), 11 mm (200 µg/mL), 10 mm (100 µg/mL); no inhibition at 50 µg/mL | - | ZnO NPs release Zn²⁺ ions that penetrate fungal cells, increasing cell wall permeability, disrupting cellular metabolism, causing ribosome disassembly, protein denaturation, nucleic acid damage, electron transport chain disruption, leakage of cytoplasmic contents, and ultimately fungal cell death. | - | - | Green-synthesized ZnO NPs produced using *Pseudomonas putida* exhibited significant concentration-dependent antifungal activity against clinical *Candida albicans* isolates from leukemia patients, highlighting their potential as an eco-friendly alternative antifungal agent. |
| (Padalia and Chanda, 2017) | Broth microdilution MIC assay, time-kill assay, agar well diffusion assay (clinical isolates) | MIC: 1.25 mg/mL against *C. albicans* and *C. glabrata*; 10 mg/mL against *C. neoformans*. MFC not reported. | - | ZnO NPs induced ROS generation (hydroxyl radicals, superoxide, singlet oxygen, nitric oxide), increased membrane permeability, disrupted membrane lipids and enzymes, caused DNA damage, and ultimately led to fungal cell death. Smaller nanoparticles exhibited greater antifungal activity because of their higher surface area. | - | Cytotoxicity was evaluated on HeLa cells. ZnO NPs showed dose-dependent cytotoxicity, reducing cell viability from approximately 89% to 39% as concentration increased (2-200 µg/mL). | Green-synthesized ZnO nanoparticles from *Ziziphus nummularia* exhibited potent antifungal activity against both reference strains and multidrug-resistant clinical *Candida* isolates, outperforming conventional azole antifungals. Their small particle size contributed to enhanced antifungal efficacy, suggesting that these eco-friendly ZnO NPs are promising candidates for treating drug-resistant candidiasis. |
| (Reda and Fayed, 2023) | CLSI-modified broth microdilution assay (liquid broth turbidity method) | MIC₅₀: CDC-B11903: 40.93 (ZC0), 77.51 (ZC10), 44.76 (ZC90) µg/mL; CDC-CAU9: 110.8 (ZC0), 167.6 (ZC10), 101.9 (ZC90) µg/mL; MFC not reported | - | ZnO NPs are internalized by *C. auris*, releasing Zn²⁺ ions that generate ROS, disrupt zinc-dependent proteins, damage lipids and proteins, interfere with metabolic pathways, impair DNA replication, and ultimately kill fungal cells | - | - | Calcium-doped ZnO ceramic nanoparticles synthesized by the sol-gel method exhibited potent antifungal activity against MDR *C. auris*. The nanoparticles were stable (negative zeta potential), showed nanoscale size with good crystallinity, and represent a promising alternative to conventional antifungal drugs against drug-resistant *C. auris* |
| (Sharma and Ghose, 2015) | Disc diffusion susceptibility assay (zone of inhibition) | MIC/MFC not reported; Zone of inhibition: 1.5 ± 0.1 mm (5 mg/mL), 5.3 ± 0.2 mm (10 mg/mL), 8.6 ± 0.4 mm (15 mg/mL), 11.4 ± 0.2 mm (20 mg/mL) | - | ZnO NPs generate ROS, including hydrogen peroxide, superoxide radicals, and hydroxyl radicals, causing fungal cell membrane damage, reduced enzymatic activity, and fungal cell death. Higher nanoparticle concentrations generate more ROS, resulting in greater antifungal activity. | - | - | Chemically synthesized ZnO NPs exhibited concentration-dependent antifungal activity against *C. albicans*. Nanoparticles with smaller size and higher surface area exhibited stronger antifungal activity, highlighting their potential as alternative antifungal agents. |
| (Shoeb et al., 2013) | Agar disc diffusion assay, in vitro killing assay (CFU), growth kinetics assay, SEM analysis, and intracellular ROS assay | MIC ≈ 29.7 µg/mL; almost complete growth inhibition at 34.1 µg/mL; MFC not reported | - | ZnO NPs entered fungal cells by diffusion/endocytosis, generated intracellular ROS (•OH, ¹O₂, O₂•⁻) and released Zn²⁺ ions, causing oxidative stress, mitochondrial dysfunction, DNA damage, membrane disruption, cavity formation, and fungal cell death. ROS scavenger (histidine) abolished antifungal activity, confirming ROS-mediated killing. | - | The synthesized ZnO NPs were reported to be biocompatible and biosafe at low concentrations; however, no direct cytotoxicity assay was performed in this study. | Egg albumen-mediated green-synthesized ZnO NPs exhibited potent dose-dependent anti*Candida*l activity against MDR *C. albicans* through ROS-mediated oxidative stress, demonstrating their potential as safe alternative antifungal nanomaterials. |
| (Souza et al., 2020) | Broth microdilution assay (MIC), AFM morphological analysis, and hemolysis assay | MIC: *C. parapsilosis* = 15.65 µg/mL (best activity); *C. krusei* = 62.25 µg/mL; *C. albicans* = 500 µg/mL; *C. tropicalis* = 1000 µg/mL; MFC not reported | - | ZnO NPs likely generate ROS and release Zn²⁺ ions, leading to disruption of the cell wall and plasma membrane, increased surface roughness, leakage of intracellular contents, and fungal cell lysis. Residual polysaccharides may also interfere with β-1,3-D-glucan synthase, impairing fungal cell wall synthesis. | - | Excellent biocompatibility; no hemolytic activity observed, maintaining 100% red blood cell viability across all tested concentrations | Green-synthesized cashew gum-capped ZnO NPs exhibited potent antifungal activity, particularly against the emerging pathogen *C. parapsilosis* (MIC: 15.65 µg/mL), along with excellent biocompatibility, making them promising *Candida*tes for antifungal coatings and therapeutic applications |
| (Yassin et al., 2023a) | Agar well diffusion assay, broth microdilution (MIC/MFC), checkerboard assay (FICI), SEM, antioxidant assay, MTT cytotoxicity assay | MIC = 100 µg/mL; MFC = 200 µg/mL for the tested *Candida* spp. | - | ZnO nanoparticles generated reactive oxygen species (ROS), released Zn²⁺ ions, damaged fungal cell membranes, oxidized proteins, lipids and DNA, induced oxidative stress, and caused apoptosis of fungal cells. | Yes. Significant synergistic activity was observed with fluconazole against *C. albicans* (FICI = 0.38), nystatin against *C. glabrata* (FICI = 0.38) and *C. tropicalis* (FICI = 0.50), and terbinafine against *C. glabrata* (FICI = 0.25). Additive effects were observed with clotrimazole and itraconazole in some strains, whereas several combinations showed no interaction. | The biogenic ZnO nanoparticles exhibited good biocompatibility toward normal WI-38 fibroblast cells, with lower toxicity than to cancer cells, indicating suitability for biomedical applications. | Green-synthesized *Salvia officinalis*-mediated ZnO nanoparticles exhibited potent antifungal activity against nosocomial *Candida* pathogens and significantly enhanced the efficacy of several conventional antifungal drugs, particularly terbinafine, nystatin, and fluconazole. These findings support the use of biogenic ZnO nanoparticles as promising nano-adjuvants for managing drug-resistant candidiasis. |
| (Yassin et al., 2023b) | Disk diffusion assay, broth microdilution (MIC/MFC), checkerboard synergy assay (FICI), SEM, MTT cytotoxicity assay | MIC = 20 µg/mL; MFC = 40 µg/mL (against *C. tropicalis*, the most susceptible isolate) | - | ZnO nanoparticles exerted antifungal activity through the generation of reactive oxygen species (ROS), disruption of cell walls and membranes, impairment of proteins and enzymes, DNA damage, and interference with the fungal antioxidant defense system, resulting in fungal cell death. | Yes. Strong synergistic interactions were observed with several commercial antifungal drugs. The greatest synergy was reported with terbinafine against *C. tropicalis*. At the same time, additive and synergistic effects were also observed with fluconazole, ketoconazole, itraconazole, nystatin, and amphotericin B, depending on the *Candida* species. | ZnO nanoparticles exhibited acceptable biocompatibility. The MTT assay showed lower toxicity toward normal WI-38 human lung fibroblast cells than toward cancer cells (MCF-7), indicating potential safety for biomedical applications. | Green tea-mediated ZnO nanoparticles exhibited potent anticandidal activity against nosocomial *Candida* species and significantly enhanced the efficacy of several conventional antifungal agents. Their small particle size, eco-friendly synthesis, broad-spectrum antifungal activity, and favorable biocompatibility make them promising nano-adjuvants for treating candidiasis and combating antifungal resistance. |
| (Yassin et al., 2025a) | Disc diffusion assay, Broth microdilution (MIC), MFC determination, SEM morphology analysis | For *C. tropicalis*: MIC = 100 µg/mL; MFC = 200 µg/mL | - | ZnO NPs penetrate fungal cells by diffusion/endocytosis, generate ROS and Zn²⁺ ions, disrupt mitochondrial activity, damage DNA, proteins, lipids, and membrane-bound enzymes, causing membrane rupture and fungal cell death. SEM confirmed wrinkled membranes, pore formation, and cell wall damage | Yes. Strong synergistic activity with fluconazole, highest against *C. glabrata*, followed by *C. parapsilosis*; moderate synergy against *C. albicans* and *C. tropicalis*. | Low cytotoxicity toward WI-38 normal lung fibroblast cells; IC₅₀ = 723.86 µg/mL, approximately 7-fold higher than the MIC, indicating good biocompatibility. | Green-synthesized ZnO NPs exhibited potent antifungal activity against multiple *Candida* species. They significantly enhanced the efficacy of fluconazole against drug-resistant strains, demonstrating potential as a safe adjunctive antifungal therapy. |
| (Yassin et al., 2025b) | Kirby-Bauer disk diffusion assay, broth microdilution (MIC/MFC), checkerboard synergy assay (FICI), SEM, antioxidant assay, MTT cytotoxicity assay | MIC = 100 µg/mL; MFC = 200 µg/mL against *C. tropicalis* | - | ZnO NPs penetrated fungal cells, generated reactive oxygen species (ROS), released Zn²⁺ ions, disrupted mitochondrial function, damaged DNA, proteins, enzymes and cell membranes, leading to fungal cell death. | Yes. ZnO NPs showed the strongest synergistic effect with nystatin (FICI = 0.38 for *C. albicans* and 0.25 for *C. tropicalis*). Additive effects were also observed with fluconazole and ketoconazole, while antagonism was observed with terbinafine against *C. tropicalis*. | ZnO NPs exhibited selective cytotoxicity. IC₅₀ = 145.2 µg/mL against HUH7 hepatoma cells and IC₅₀ = 237.6 µg/mL against WI38 normal lung fibroblasts, indicating good biocompatibility at antifungal concentrations. | Green-synthesized *L. sativum*-mediated ZnO nanoparticles demonstrated potent antifungal activity against drug-resistant *Candida* species. Their combination with nystatin produced marked synergistic effects, suggesting their potential as an effective nano-adjuvant therapy for resistant candidiasis. The nanoparticles also showed favorable biocompatibility and antioxidant activity, supporting their potential for future biomedical applications. |

**Reference**

Abedzadeh, H.A., Dakhili, M., Saghazadeh, M., Aghaei, S. and Nazari, R. 2022. Changes of gene expression in Candida albicans isolates from vaginal infections by effects of zinc oxide nanoparticles and fluconazole.

Abou-Dobara, M.I., Baka, Z.A., El-Salamony, S.M. and El-Zahed, M.M. 2025. In Vitro anticandidal efficiency of newly synthesized zinc oxide/chitosan/vancomycin nanocomposite using Bacillus Licheniformis compared to fluconazole.

Ahmadpour Kermani, S., Salari, S. and Ghasemi Nejad Almani, P. 2021. Comparison of antifungal and cytotoxicity activities of titanium dioxide and zinc oxide nanoparticles with amphotericin B against different Candida species: In vitro evaluation. Journal of clinical laboratory analysis 35(1), e23577.

Albuquerque, L.A., Sangappa, S.B., Srinivasan, A., Archer, A. and Rao, R. 2023. Efficacy of chemically and biologically synthesized zinc oxide nanoparticles incorporated in soft denture liner against candida albicans: a comparative in vitro study. World Journal of Dentistry 14(10), 851-859.

Alves, M.M., Cunha, D.V., Santos, C.F., Mira, N.P. and Montemor, M.F. 2018. Microstructured ZnO-rod like coating prevents biofilm formation prompted by pathogenic Candida spp. Ceramics International 44(4), 4467-4472.

Anitha, R., Singaravel, V. and Vinoth, J. 2023. Zinc oxide nanoparticle inhibits the biofilm formation of Candida glabrata: a sustainable approach to use an agro-waste of cashew apple juice. Biomass Conversion and Biorefinery 13(16), 15083-15093.

Aslani, P., Roudbar, M.S. and Roudbary, M. 2018. Novel formulated zinc oxide nanoparticles reduce Hwp1 gene expression involved in biofilm formation in Candida albicans with minimum cytotoxicity effect on human cells.

Dananjaya, S., Kumar, R.S., Yang, M., Nikapitiya, C., Lee, J. and De Zoysa, M. 2018. Synthesis, characterization of ZnO-chitosan nanocomposites and evaluation of its antifungal activity against pathogenic Candida albicans. International journal of biological macromolecules 108, 1281-1288.

Das, B., Khan, M.I., Jayabalan, R., Behera, S.K., Yun, S.-I., Tripathy, S.K. and Mishra, A. 2016. Understanding the antifungal mechanism of Ag@ ZnO core-shell nanocomposites against Candida krusei. Scientific reports 6(1), 36403.

Djearamane, S., Xiu, L.-J., Wong, L.-S., Rajamani, R., Bharathi, D., Kayarohanam, S., De Cruz, A.E., Tey, L.-H., Janakiraman, A.K. and Aminuzzaman, M. 2022. Antifungal properties of zinc oxide nanoparticles on Candida albicans. Coatings 12(12), 1864.

Fayed, B., El-Sayed, H.S., Luo, S. and Reda, A.E. 2025. Comparative evaluation of biologically and chemically synthesized zinc oxide nanoparticles for preventing Candida auris biofilm. BioMetals 38(3), 817-830.

Fozouni, L. and Tahaei, M. 2023. Anticandidal effects of zinc oxide nanoparticles on fluconazole-resistant candida isolates causing diarrhea in calves, in vitro. Archives of Razi Institute 78(1), 499.

Hameed, A.S.H., Karthikeyan, C., Kumar, V.S., Kumaresan, S. and Sasikumar, S. 2015. Effect of Mg2+, Ca2+, Sr2+ and Ba2+ metal ions on the antifungal activity of ZnO nanoparticles tested against Candida albicans. Materials Science and Engineering: C 52, 171-177.

Hosseini, S.S., Ghaemi, E. and Koohsar, F. 2018. Influence of ZnO nanoparticles on Candida albicans isolates biofilm formed on the urinary catheter. Iranian journal of microbiology 10(6), 424.

Hosseini, S.S., Ghaemi, E., Noroozi, A. and Niknejad, F. 2019. Zinc oxide nanoparticles inhibition of initial adhesion and ALS1 and ALS3 gene expression in Candida albicans strains from urinary tract infections. Mycopathologia 184(2), 261-271.

Hosseini, S.S., Joshaghani, H., Shokohi, T., Ahmadi, A. and Mehrbakhsh, Z. 2020. Antifungal activity of ZnO nanoparticles and nystatin and downregulation of SAP1-3 genes expression in fluconazole-resistant Candida albicans isolates from vulvovaginal candidiasis. Infection and drug resistance, 385-394.

Jalal, M., Ansari, M.A., Ali, S.G., Khan, H.M. and Rehman, S. 2018. Anticandidal activity of bioinspired ZnO NPs: effect on growth, cell morphology and key virulence attributes of Candida species. Artificial cells, nanomedicine, and biotechnology 46(sup1), 912-925.

Joshi, K.M., Shelar, A., Kasabe, U., Nikam, L.K., Pawar, R.A., Sangshetti, J., Kale, B.B., Singh, A.V., Patil, R. and Chaskar, M.G. 2022. Biofilm inhibition in Candida albicans with biogenic hierarchical zinc-oxide nanoparticles. Biomaterials Advances 134, 112592.

Jothiprakasam, V., Sambantham, M., Chinnathambi, S. and Vijayaboopathi, S. 2017. Candida tropicalis biofilm inhibition by ZnO nanoparticles and EDTA. Archives of Oral Biology 73, 21-24.

Katafa, A.J. and Hamid, M.K. 2020. Influence of zno nanoparticles on Candida Albicans of human male pleural fluid. Iraqi Journal of Science, 540-549.

Maniah, K. 2023. Anticandidal effectiveness of greenly synthesized zinc oxide nanoparticles against candidal pathogens. Journal of Environmental Science and Health, Part A 58(14), 1097-1110.

Mathew, D., Thomas, B., Sudheep, N., Nair, S. and Radhakrishnan, E. 2025. Mangifera indica phytochemical enriched ZnO-Amphotericin B Nanoconjugates: Enhanced hydrophilicity, reduced toxicity and pH-sensitive release for targeted anti-Candida therapy. Advanced Powder Technology 36(6), 104895.

Metwally, R.S., MH Kheiralla, Z., M Ashour, S. and S Zaki, S. 2022. Anticandidal activity of green synthesized zinc oxide nanoparticles using lemon peel extract. Journal of Scientific Research in Science 39(2), 62-81.

Mohammed, I.S., Nafea, M.H., Jawad, R.S., Abd, D.A. and Aljoubory, H.M. 2024. Evaluation of the effect of green synthesis of Zinc oxide nanoparticles on Candida albicans. Journal of Bioscience and Applied Research 10(3), 518-529.

Padalia, H. and Chanda, S. 2017. Characterization, antifungal and cytotoxic evaluation of green synthesized zinc oxide nanoparticles using Ziziphus nummularia leaf extract. Artificial cells, nanomedicine, and biotechnology 45(8), 1751-1761.

Reda, A. and Fayed, B. 2023. The synthesis of calcium doped zinc oxide ceramic nanoparticles via sol–gel effective against the emerging multidrug-resistant Candida auris. Journal of the Australian Ceramic Society 59(5), 1315-1323.

Sharma, R.K. and Ghose, R. 2015. Synthesis of zinc oxide nanoparticles by homogeneous precipitation method and its application in antifungal activity against Candida albicans. Ceramics International 41(1), 967-975.

Shoeb, M., Singh, B.R., Khan, J.A., Khan, W., Singh, B.N., Singh, H.B. and Naqvi, A.H. 2013. ROS-dependent anticandidal activity of zinc oxide nanoparticles synthesized by using egg albumen as a biotemplate. Advances in Natural Sciences: Nanoscience and Nanotechnology 4(3), 035015.

Souza, J.M.T., de Araujo, A.R., de Carvalho, A.M.A., Amorim, A.d.G.N., Daboit, T.C., de Almeida, J.R.d.S., da Silva, D.A. and Eaton, P. 2020. Sustainably produced cashew gum-capped zinc oxide nanoparticles show antifungal activity against Candida parapsilosis. Journal of Cleaner Production 247, 119085.

Yassin, M.T., Al-Otibi, F.O., Al-Askar, A.A. and Elmaghrabi, M.M. 2023a. Synergistic anticandidal effectiveness of greenly synthesized zinc oxide nanoparticles with antifungal agents against nosocomial candidal pathogens. Microorganisms 11(8), 1957.

Yassin, M.T., Al-Otibi, F.O., Maniah, K., Mohamed, S., Hasan, K.A., Negi, A. and AbdelGawwad, M.R. 2025a. Synergistic antifungal efficiency of Eco-Friendly synthesized zinc oxide nanoparticles in combination with fluconazole against Drug-Resistant candidal strains. Polish Journal of Environmental Studies 34(2), 1851-1865.

Yassin, M.T., Elgorban, A.M., Al-Askar, A.A., Sholkamy, E.N., Ameen, F. and Maniah, K. 2023b. Synergistic anticandidal activities of greenly synthesized ZnO nanomaterials with commercial antifungal agents against candidal infections. Micromachines 14(1), 209.

Yassin, M.T., Mohamed, S., Al-Otibi, F.O., Maniah, K. and AbdelGawwad, M.R. 2025b. Synergistic antifungal activity of lepidium sativum ZnO nanoparticles and nystatin against resistant candida species. Scientific Reports 15(1), 34650.
